# Human Tendon‐on‐Chip: Unveiling the Effect of Core Compartment‐T Cell Spatiotemporal Crosstalk at the Onset of Tendon Inflammation

**DOI:** 10.1002/advs.202401170

**Published:** 2024-09-11

**Authors:** Syeda M. Bakht, Alberto Pardo, Manuel Gomez‐Florit, David Caballero, Subhas C. Kundu, Rui L. Reis, Rui M. A. Domingues, Manuela E. Gomes

**Affiliations:** ^1^ 3B's Research Group I3Bs – Research Institute on Biomaterials Biodegradables and Biomimetics University of Minho Headquarters of the European Institute of Excellence on Tissue Engineering and Regenerative Medicine AvePark – Parque de Ciência e Tecnologia Zona Industrial da Gandra Barco Guimarães 4805‐017 Portugal; ^2^ ICVS/3B's – PT Government Associate Laboratory Braga/Guimarães Portugal; ^3^ Colloids and Polymers Physics Group Particle Physics Department Materials Institute (iMATUS) and Health Research Institute (IDIS) University of Santiago de Compostela Santiago de Compostela 15782 Spain; ^4^ Health Research Institute of the Balearic Islands (IdISBa) Palma 07010 Spain; ^5^ School of Medicine and Biomedical Sciences (ICBAS), Unit for Multidisciplinary Research in Biomedicine (UMIB) University of Porto Rua Jorge Viterbo Ferreira 228 Porto 4050‐313 Porto Portugal

**Keywords:** anisotropy, cell migration, compartmentalization, in vitro models, magnetic hydrogels, tendon‐on‐chip, tissue engineering

## Abstract

The lack of representative in vitro models recapitulating human tendon (patho)physiology is among the major factors hindering consistent progress in the knowledge‐based development of adequate therapies for tendinopathy.Here, an organotypic 3D tendon‐on‐chip model is designed that allows studying the spatiotemporal dynamics of its cellular and molecular mechanisms.Combining the synergistic effects of a bioactive hydrogel matrix with the biophysical cues of magnetic microfibers directly aligned on the microfluidic chip, it is possible to recreate the anisotropic architecture, cell patterns, and phenotype of tendon intrinsic (core) compartment. When incorporated with vascular‐like vessels emulating the interface between its intrinsic‐extrinsic compartments, crosstalk with endothelial cells are found to drive stromal tenocytes toward a reparative profile. This platform is further used to study adaptive immune cell responses at the onset of tissue inflammation, focusing on interactions between tendon compartment tenocytes and circulating T cells.The proinflammatory signature resulting from this intra/inter‐cellular communication induces the recruitment of T cells into the inflamed core compartment and confirms the involvement of this cellular crosstalk in positive feedback loops leading to the amplification of tendon inflammation.Overall, the developed 3D tendon‐on‐chip provides a powerful new tool enabling mechanistic studies on the pathogenesis of tendinopathy as well as for assessing new therapies.

## Graphical abstract

This study develops a novel Human 3D Tendon‐on‐Chip as representative in vitro model for studying cellular crosstalk in tendon health and disease. The design of this bioengineered microphysiological system recreates the tendon architecture and cellular patterns. It has been applied for studying multicellular signaling dynamics, including crosstalk of tendon core with endothelial and T‐cell responses under inflammatory environments.

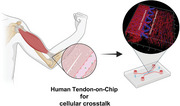

## 1 Introduction

Tendons are multicellular connective tissues with a highly‐ordered hierarchical structure that strongly dictates their complex physicochemical properties and functionalities.^[^ ^1^ ^]^ Tendinopathy encompasses all diseases associated with the impaired function of tendon tissues resulting in swelling or activity‐related pain, among other symptoms or injury in severe cases.^[^ ^2^, ^3^ ^]^ Degenerative tendon injuries often result from repetitive strain induced microdamage when the normal thresholds (overuse) is exceeded.^[^ ^4^ ^]^ Upon injury, activation of inflammatory mechanisms dysregulates tissue homeostasis. Although this early inflammatory response is essential to promote healing, the uncontrolled perpetuation of this mechanism often leads to detrimental consequences in affected tissues.^[^ ^4^, ^5^ ^]^ Current knowledge on inflammatory mechanisms specifically involved in tendinopathy is very limited, mainly due to the lack of accurate in vitro tendon models that can faithfully recreate the complex cellular interaction between somatic cells and the immune system,^[^ ^6^, ^7^ ^]^ as well as the role of molecular inflammatory mediators that dictate the progress of tendon healing processes.^[^ ^2^, ^8^ ^]^

**Figure 1:**
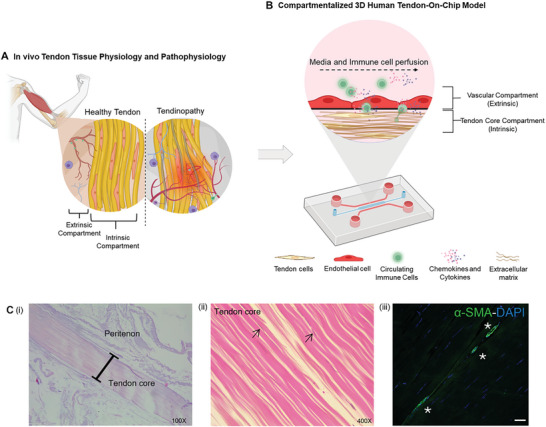
Schematic representation of the compartmentalized 3D Human Tendon‐on‐a‐Chip Model (3D‐TenOC) herein proposed for the in vitro investigation of the cellular crosstalk in tendon tissue under physiological and inflamed conditions. A) Tendon structure and pathogenesis: the intrinsic compartments of healthy tendons are formed by parallel collagen fibers arranged in neat and organized patterns. Blood vessels and nerves are responsible for supplying nutrients and sensory inputs to the tendon. These, together with a population of resident immune cells, form the extrinsic compartment in healthy tendons. During the progression of tendinopathy, collagen fibers become disorganized, and there is an increase in collagen III. The release of cytokines and chemokines from the injured tendon regions triggers angiogenesis and recruitment of immune cells and affects adjacent healthy tissues. B) The 3D‐TenOC model recapitulates the key components of the compartmentalized tendon intrinsic and extrinsic compartments, which was here applied to investigate in vitro the cellular crosstalk at the onset of tissue inflammatory responses. C) Histological sections of human cadaveric plantar flexor tendon (i) stained with Alcian Blue/Eosin showing the tendon core surrounded by peritenon, (ii) higher magnification image of tendon core (stained with Hematoxylin and Eosin), where black arrows highlight nucleus (purple), and (iii) DAPI (blue) and anti‐alpha smooth muscle actin (green) immunofluorescent staining of tendon core, where asterisk identifies capillaries (scale bar: 20 µm).

Tendon tissue can be categorized into two basic compartments: an “intrinsic compartment” comprising uniaxially aligned tendon cells connected into rows in head‐to‐tail fashion embedded into a dense extracellular matrix (ECM) rich in anisotropically aligned collagen fibers; and an “extrinsic compartment” hosting resident immune cells and with connections from the vascular and nervous system (**Figure** **1**A).^[^ ^4^, ^9^ ^]^ Despite the recognized importance of the interaction mechanisms between these two compartments and their coordination for tissue functionality, this remains to be fully elucidated.^[^ ^10^, ^11^, ^12^ ^]^

The progression of tendinopathy lead to tissue alterations^[^ ^13^ ^]^ characterized by increased disorganization of fibrillar collagen structure, the accumulation of a mucoid ground substance, and the arbitrary increase in vascularity,^[^ ^14^, ^15^ ^]^ followed by complex alterations in tissue innervation.^[^ ^12^, ^16^ ^]^ Increased presence of resident and infiltrating immune cells, along with a cascade of inflammatory cytokines, is typically found in tendinopathic tissues, being hypothesized that the crosstalk between stromal and immune tendon cells can eventually dictate if damaged tissues follow the path toward regeneration or degenerate into fibrosis.^[^ ^6^, ^7^, ^8^, ^15^ ^]^ Importantly, these alteration may affect not only the injury site but also the adjacent normal portions of the tendon, leading to what is called “reactive‐on‐degenerative tendinopathy”.^[^ ^17^ ^]^ A main barrier to the development of effective therapies for promoting tendon regeneration is the current limited understanding of the cellular signaling occurring during healing, which is governed by the complex interplay of multiple biological factors.^[^ ^2^ ^]^ Although recent studies consistently highlight the key role of both innate and adaptive immune system in tendon hemostasis and degeneration, the cellular mechanisms driving these (patho)physiological processes are just beginning to be elucidated.^[^ ^18^ ^]^ While most of the research in this field has centered on deciphering the molecular and inflammatory cellular signaling mechanisms related to the innate immune cells (particularly macrophages),^[^ ^19^ ^]^ little is known about the actual role of adaptive immune cells in tendon health and disease. In case of acute insult, e.g., excessive mechanical stress, tenocytes release inflammasome activated cytokines such as IL‐1β, which in turn can promote the recruitment of proinflammatory cells that perpetuate the inflammatory cycle by releasing pro‐inflammatory molecules such as TNF‐α, IL‐6, IL‐17.^[^ ^20^ ^]^ Soluble antigens and cytokines derived from damaged tissue are known to trigger the activation of T cells, which subsequently migrate to the injury site where they exert their immunomodulatory functions.^[^ ^21^ ^]^ Interestingly, the direct cell‐cell contact of tenocytes with T cell in 2D co‐cultures seems to promote their mutual activation, leading to further upregulation of proinflammatory cues and creating an amplifying feedback loop that may be related with the establishment of chronic tendon inflammation.^[^ ^22^ ^]^ These studies suggest that manipulation of adaptive immunity having T cells as targets might be a potential therapeutic strategy for resolving inflammation and promoting tendon healing. Advances in this field will however require further investigation of T cell‐mediated effects in tendon hemostasis and disease progression.

Animal^[^ ^23^, ^24^, ^25^ ^]^ and explant‐based models^[^ ^26^, ^27^, ^28^ ^]^ have been typically used for studying tendinopathy and its progression under complex physiological environments, as well as to analyze the effects of specific factors of the tendon niche, such as the impact of specific cell types on tissue physiology.^[^ ^29^, ^30^ ^]^ However, despite their advantages,^[^ ^26^, ^31^ ^]^ and in addition to its cost and increased societal pressure to reduce animal experimentation, animal testing often fails to faithfully recapitulate human‐like responses due to physiological differences between species, while explants are, e.g., associated with time constraints on their *ex vivo* maintenance or with limited accuracy on recapitulating human diseased conditions.^[^ ^7^ ^]^ Relevant in vitro models of human tendon health and diseases are therefore critical for enabling consistent progress in this field.

Ideally, a representative in vitro tendon model must recreate key features of native tissues, namely (i) the hierarchical and highly‐anisotropic fibrous architecture of tendon ECM, (ii) the heterogeneity and structural organization of tendon cells population, and (iii) the physiological signaling of tendon niche (Figure 1).^[^ ^7^, ^32^ ^]^ As an alternative to a typical 2D well plate and transwell membrane co‐culturing systems,^[^ ^22^ ^]^ several bioengineering strategies have been proposed in order to reproduce the hierarchical anisotropic organization of tendon architecture.^[^ ^33^, ^34^, ^35^ ^]^ For example, Snedeker et al. investigated how fibrous substrate‐driven cell morphology can influence cell sensitivity to proinflammatory signaling by co‐culturing tenocytes with macrophages.^[^ ^6^ ^]^ Our team has further developed composite living fibers with different 3D fibrillar architectures modeling some key hallmark responses of tenocytes in a healthy and diseased tendon stroma.^[^ ^36^ ^]^ However, these simplistic models do not recreate neither tissue compartmentalization structures nor their cell patterns, which have a considerable impact on cell signaling and are required to investigate the multicellular spatiotemporal crosstalk occurring under dynamic tendon mimetic conditions.

Organs‐on‐a‐chip (OoC) technology has been gaining increased interest for in vitro modeling applications.^[^ ^37^, ^38^ ^]^ OoC are typically built on microfluidic devices housing multiple cell types and ECM components (natural or synthetic), arranged in interconnected compartments to reproduce specific tissue‐like functions. The increased physiological relevance of these systems, compared not only to planar cell culture but also to animal models, has been widely demonstrated in recent years.^[^ ^39^ ^]^ However, although numerous OoC systems have been developed for a wide range of tissues and organs,^[^ ^39^ ^]^ to our knowledge, tendon OoC systems have not been established so far.

Here, we developed a compartmentalized human 3D Tendon‐on‐Chip (3D‐TenOC) that emulates the essential structural and architectural features of tendon tissues. A main challenge solved by the proposed concept is how to directly reconstruct on a chip the 3D hierarchical fibrous organization of native tendons while integrating relevant cell populations that mimic the spatial and temporal resolution of their in vivo microenvironment. By combining microfluidic platforms with magnetically‐assisted hydrogel microstructuring, we produced microphysiological systems consisting of a compartmentalized tendon‐like core compartment with vasculature (from the extrinsic compartment), where the crosstalk with circulating T cells at the onset of tissue inflammation can be effectively studied (Figure 1B). This tissue‐mimetic co‐culture system enabled to identify alterations in the gene expression and cytokine profiles of core compartment tenocytes as a consequence of the established crosstalk with other relevant tendon cell populations under different physiological and inflamed conditions. Overall, obtained results suggest that the proposed 3D‐TenOC platform can serve as a valuable preclinical tool for screening the effects of intercellular communication among different cell populations of tendon tissues, as well as their roles in both health and progression to disease states.

## 2 Results and Discussion

### 2.1 Recreating the Intrinsic and Extrinsic Tendon Compartments in a 3D Human Tendon‐On‐Chip Model (3D‐TenOC)

To study tendon physiology and the mechanisms of its disease pathology, we need models representing the cellular and architectural patterns of the tissue. In healthy tendons, blood vessels are arranged longitudinally in the epitenon, and pass through the endotenon surrounding the fiber bundles (fascicles).^[^ ^40^ ^]^ In this work, we aimed at designing a compartmentalized microfluidic chip device where the fundamental characteristics of the intrinsic core compartment and its vascular system were recreated, building a microphysiological system that allows interrogating the complex multicellular crosstalk occurring under different tendon microenvironments. Various design approaches were combined sequentially to meet the desired 3D‐TenOC characteristics, namely, to engineer a hydrogel matrix that can control cell organization and sustain its tenogenic phenotype in the core compartment and recapitulate the vascular vessels from the extrinsic compartment.

#### 2.1.1 Engineering the Core Compartment

**Figure 2:**
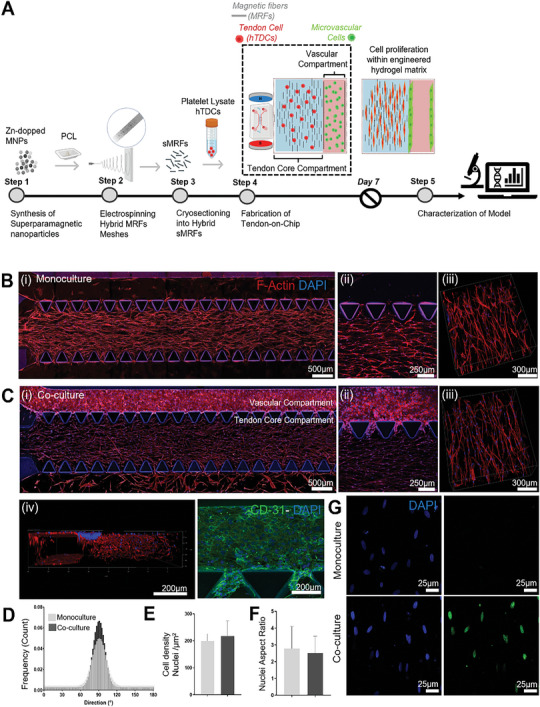
Fabrication and characterization of 3D‐TenOC. A) Schematics representing the workflow development of 3D‐TenOC. The process starts with the synthesis of zinc‐doped magnetic nanoparticles (Zn‐MNPs) and their incorporation in the structure of electrospun polycaprolactone (PCL) microfibers. Then, the electrospun membranes are cryosectioned into short magnetically‐responsive microfibers (sMRFs). For the fabrication of the intrinsic compartment, human tendon cells (hTDCs) and sMRFs are embedded into platelet lysate (PL) and loaded into the central compartment of the microfluidic chip under the application of uniform magnetic fields. After the magnetically‐induced alignment of sMRFs and gelation of PL, the magnetic field is removed, and vascular cells are introduced into the side channel. The cells are kept in culture within the engineered hydrogel matrix for 7 days, and then 3D‐TenOC is evaluated. Confocal laser microscopy (CLM) images showing actin cytoskeleton alignment (red) and nuclei (blue) in B) monoculture (hTDCs seeded in the central compartment), and C) co‐culture systems (hTDCs and vascular cells seeded in the central and side channels, respectively); (i) tile scan image, (ii) magnification of central and side channel, (iii) magnification of central channel, iv) 3D reconstruction CLM images within co‐culture fabricated chip where vascular cells inside the lateral compartment organize into a tubular monolayer and v) CLM images co‐culture model with CD 31 (green) and nuclei (blue) staining for vascular forming cell‐cell contact (scale bar: 500 µm, 250 µm, 300 µm 200 µm and 100 µm respectively). Plot of directionality analysis of cells within the central compartment E) Estimation of cell density based on nuclei density by areas. F) Nuclei aspect ratio on day 7 of culture. G) Ki‐67 staining(i) monoculture (ii) coculture (scale bar: 25 µm). Statistical differences derived from confocal microscopy images of central compartment; monoculture: n = 9, co‐culture: n = 13, ns = not significant and **p < 0.005, determined by unpaired two‐tailed t‐test.

The first step of our 3D‐TenOC design required to devise a feasible strategy for fabricating a tendon‐like core tissue directly on the chip. We have previously developed bioengineered 3D living fibers consisting of anisotropic electrospun polycaprolactone (PCL) fibers cores coated with platelet lysate (PL) hydrogel shell encapsulating human tendon cells (hTDCs).^[^ ^36^ ^]^ We have shown that the synergy between the biophysical cues of PCL fibers with the biochemical signaling of PL components^[^ ^35^, ^41^, ^42^ ^]^ can effectively drive hTDCs tenogenesis and that this xeno‐free microsystem can be explored for modeling different scenarios of tendon stroma microenvironment in vitro. Building on this concept, here we proposed a new approach to translate its design principle into a microfluidic device. To enable their loading through the central chip chamber inlet, short magnetically‐responsive microfibers (sMRFs) were first produced and then suspended in a PL solution carrying hTDCs. Once in the chamber, sMRFs can be remotely aligned to generate a tendon‐biomimetic anisotropic fibrous architecture that dictates cell growth and orientation by contact guidance mechanism (**Figure** **2**).^[^ ^43^ ^]^

The fabrication of the intrinsic compartment of the 3D‐TenOC was carried out in several steps, as illustrated in Figure 2A. sMRFs were synthesized following our previously established electrospinning‐microcutting protocol.^[^ ^43^ ^]^ Briefly, superparamagnetic zinc‐doped iron oxide nanoparticles (Zn‐MNPs) were prepared through a thermal decomposition route (Figure S1A, Supporting Information). The incorporation of zinc cations in the crystalline lattice of iron oxide nanoparticles has demonstrated to be an efficient strategy for increasing their magnetization values.^[^ ^44^, ^45^ ^]^ This high magnetic susceptibility allows to reduce the amount of Zn‐MNPs required for providing our systems with the desired magnetic response, thus minimizing the potential cytotoxicity concerns associated with the use of high concentrations of this inorganic nanomaterials.^[^ ^46^ ^]^ Zn‐MNPs were then incorporated at 5% (w/w) concentration into electrospun PCL microfiber meshes to render them magnetically‐responsive, followed by their microcutting for obtaining sMRFs with average length of 41 ± 11 µm (Figures S1B,C; Figure S3B, Supporting Information).

To replicate the highly‐uniaxial anisotropic architecture of native tendons, sMRFs were firsts suspended in the PL solution, loaded into the central chamber of the microfluidic chip, and then aligned through the application of uniform magnetostatic fields generated by two‐parallel neodymium magnets separated 2.5 cm in a custom‐made holder (Figure S2, Supporting Information).^[^ ^43^ ^]^ Both computational simulation and experimental measurements showed the existence of a region *ca*. 1.2 cm wide between the magnets where the generated magnetic field gradient was fairly uniform (85–100 mT, Supporting Information), as illustrated in Figure S3A (Supporting Information). This uniformity is essential to ensure the alignment of the sMRFs within the chip while avoiding their aggregation/accumulation in the regions closer to magnets. Moreover, it is worth mentioning that the high magnetic power of the designed Zn‐MNPs allowed to control the orientation of the SMRFs by applying low‐strength magnetic fields, thus minimizing the potential safety risks associated with the use of intense magnetic radiations. After alignment, sMRFs orientation in the microfluidic chip chamber was fixed by gelation of PL hydrogel precursors solution (induced by thrombin and calcium chloride, Figure S3Aiii, Supporting Information).^[^ ^36^, ^47^ ^]^ After optimization of the fabrication process, hTDCs at cell density of 2.10^6^ cells mL^−1^ were suspended in the PL solution and the same loading and sMRFs alignment strategy was followed to fabricate the cellularized core compartment (see section 2.1.3).

#### 2.1.2 Recapitulating Vascular Vessels from Extrinsic Compartment

Physiologically relevant tendon in vitro models should consider all the different cell populations with known implications on tendon health and disease states.^[^ ^48^ ^]^ While tendon exhibit low cellularity and hypo‐vascularity^[^ ^7^, ^11^ ^]^ in healthy state, the vascular compartment is increasingly recognized to play important roles in tissue development, homeostasis and diseased state. However, the proper functionality of mature tendon has been traditionally believed to heavily depend on synovial fluid diffusion, serving as a conduit to deliver all essential growth factors.^[^ ^49^, ^50^, ^51^ ^]^ Moreover, increased angiogenesis and neural sprouting are evident at damaged tendon sites, processes that are known to play significant roles in the modulation of the inflammatory response.^[^ ^8^, ^52^, ^53^, ^54^, ^55^ ^]^ Nevertheless, how vascular vessels impact stromal tendon cell responses has not been fully elucidated by previous studies.^[^ ^49^, ^53^ ^]^ Therefore, following the fabrication of the tendon core, we next incorporated the vascular‐like vessels on the chip. After the complete gelation of PL hydrogel matrix in the central compartment, the microfluidic chip was removed from the magnetic device and endothelial cells were injected into the lateral channel at cell density of 6.10^6^ cells mL^−1^. During the incubation period, the chip was flipped twice to guarantee even cell adhesion along the walls of the lateral channel in the microfluidic chip. After 30 minutes, fresh media was circulated to eliminate any non‐adhered cells, and the chip was then cultured for a week. After 7 days of culture, cells formed an open endothelialized microvessel‐like structure with a perfusable lumen, mimicking the vascular system existing on the tendon extrinsic compartment (Figure 2C; Figure S4B, Supporting Information).

#### 2.1.3 Engineered Hydrogel Matrix Controls Cell Orientation in Tendon Core Compartment

The extracellular topographic environment has been shown to impact basic cellular features such as adhesion, morphology, and orientation and consequently further complex cellular functions like proliferation, differentiation, and cell migration.^[^ ^56^, ^57^ ^]^ Thus, the capacity of sMRFs orientation to induce cell contact guidance alignment was evaluated. Directionality analysis of the encapsulated hTDCs was performed in both monoculture and co‐culture systems with vascular cells on day 7 of culture. Unlike cells cultured on hydrogels with randomly‐oriented sMRFs (Figure S4A, Supporting Information), in anisotropic hydrogels, actin cytoskeleton followed the orientation of sMRFs, creating uniaxial alignment and elongation of cells (Figure 2B,C), a morphological characteristic to tenocytes within tendon fascicles.^[^ ^4^ ^]^ As observed by the directionality analysis, the presence of vascular cells did not affect hTDCs’ cytoskeleton alignment (Figure 2D) nor their cell density (Figure 2E) after 7 days of culture.

Although the cell density of healthy tendon core tissue varies significantly depending on animal species and its age, specific tendon type and even the experimental method used to count cells, it typically lies between about 50 and 1000 cells mm^−2^.^[^ ^58^, ^59^, ^60^, ^61^, ^62^, ^63^, ^64^ ^]^ The cell density of our tendon core compartment is 257,4 ± 24,8 cells mm^−2^ counted in full chip thickness projection images and 52,4 ± 2,6 cells mm^−2^ in respective thin 20 µm z‐stacks. Thus, these values are fairly close of the normal cell density reported for healthy adult mammalian tissues. However, despite showing comparable cell density in both conditions, Ki‐67 staining suggest that hTDCs in co‐culture system are in a more active proliferative phase (high ratios of Ki‐67 positive cells), while it entered into a mainly quiescent state in monoculture (Figure 2G).

Previous studies have shown that external physical signals from ECM architecture can not only impact the alignment of the cell's cytoskeleton but also alter the morphology of the nucleus, which is known to regulate gene expression patterns by mechanotransduction mechanisms.^[^ ^65^ ^]^ As observed in Figure 2F, nuclei of hTDCs were co‐aligned with the main axis of the cell body and presented high aspect ratios in both monoculture and co‐culture conditions (2.8 ± 1.1 and 2.5 ± 0.8 µm, respectively), indicative of high cytoskeletal tension. Similar morphology was observed in native tenocytes that tend to elongate and orient in response to ECM fiber alignment and mechanical stress.^[^ ^66^, ^67^, ^68^ ^]^ Our findings are consistent with our previous work,^[^ ^43^ ^]^ in which we have demonstrated that sMRFs alignment in hydrogel matrices can effectively dictate cell orientation through contact guidance, recreating the ECM architecture and cellular patterns of the tendon tissues. Interestingly, results also demonstrated that the co‐culture of hTDCs with vascular cells does not have a significant impact on hTDCs morphology for the time period studied. Moreover, some infiltration of endothelial cells into the core (intrinsic compartment) can be noticed at the interface between the vascular and core compartments (Figure 2Cv), suggesting a good integration of the two cellular structures.

### 2.2 Tendon Core Phenotype Supported by the Bioengineered Microenvironment

**Figure 3:**
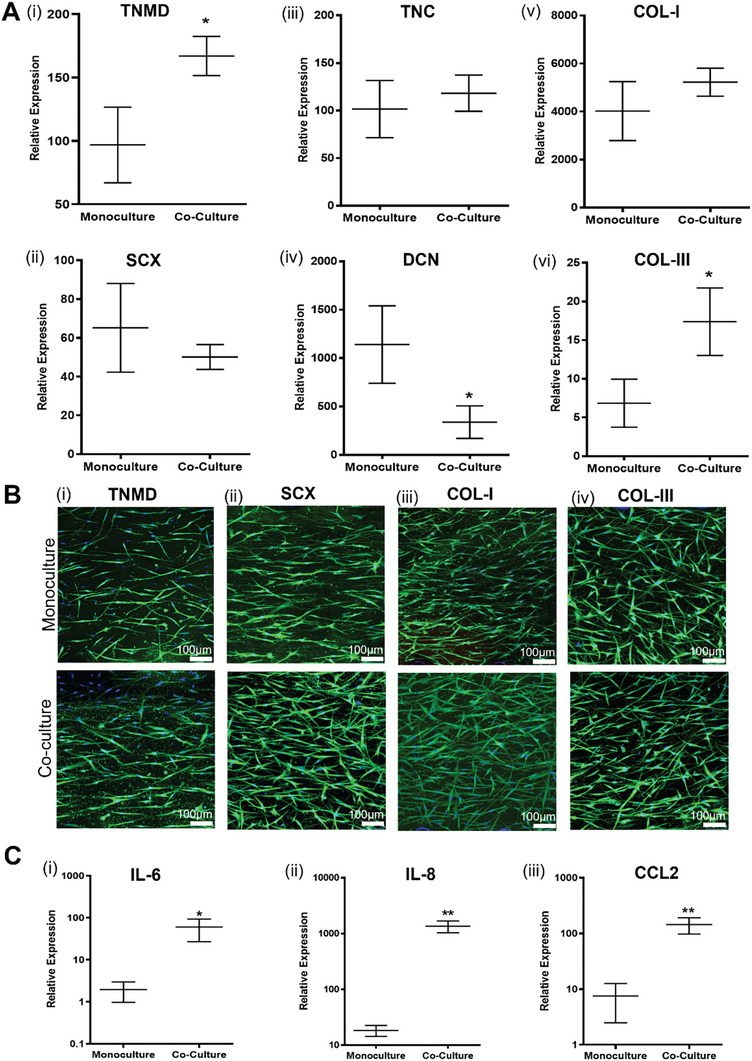
Expression of tendon‐related markers in the core compartment of the 3D‐TenOC. A) Gene expression: for the phenotype markers (i) tenomodulin (TNMD) *p < 0.05 and (ii) scleraxis (SCX); and for matrix components (iii) tenascin (TNC), (iv) decorin (DCN), (v) collagen type 1 (COL‐I), and (vi) collagen type III (COL‐III) *p < 0.05. Statistical significance was determined by an unpaired two‐tailed t‐test. B) Immunocytochemistry analysis for (i) TNMD, (ii) SCX, (iii) COL‐I and COL‐III (iv). CLM images showing fluorescent protein (green) and nuclei (blue). Scale bars: 100 µm. C) Proinflammatory gene expression in hTDCs in monoculture and co‐culture with vascular cells. Expression of interleukins (i) IL‐6, (ii) IL‐8, and (iii) C‐C Motif Chemokine Ligand 2 (CCL2). Data presented as mean ± standard deviation (n = 3), statistical difference **p < 0.005, *p < 0.05 determined by unpaired two‐tailed t‐test.

Another major difficulty of tendon in vitro modeling is the susceptibility of tenocytes to undergo fast phenotype drift during in vitro culture.^[^ ^69^ ^]^ Bioengineering strategies based on different scaffolding systems recreating the 3D architecture of tendon niche have revealed effective on either maintaining the phenotype of tenocytes in vitro and/or promoting the differentiation of stem cells toward tenogenic lineage.^[^ ^36^, ^70^, ^71^ ^]^ The potential of our 3D‐TenOC to support the tenogenic commitment of encapsulated hTDCs was evaluated by first analyzing the expression of known tendon phenotype (Scleraxis (SCX) and Tenomodulin (TNMD)) markers at both gene and protein expression level (Figure S5, Supporting Information). Scleraxis (SCX) is a transcription factor primarily expressed in tenocytes that plays a significant role in tendon development and maintenance.^[^ ^72^, ^73^ ^]^ TNMD belongs to the transmembrane glycoproteins type II family and is known to be the regulator for matrix remodeling. It is considered one of the main tendon‐related markers, providing crucial information on the capacity of tendon tissue to undergo repairing mechanisms and its ability to adapt in response to mechanical stress or injury.^[^ ^74^, ^75^ ^]^ On day 7 of culture, we observed an increase in the transcript levels of TNMD and SCX, suggesting the maintenance of a robust tenogenic phenotype (**Figure** **3**Ai‐ii), which was further confirmed at protein expression level (Figure 3Bi‐ii). Interestingly, although no significant differences were observed in the case of SCX expression profile, TNMD gene expression was significantly higher in co‐culture when compared to monoculture, a trend that is in agreement with our previous results.^[^ ^48^ ^]^ The effects of microenvironment and cell co‐culture conditions on tendon‐related ECM markers, (including collagen type I and III, tenascin, and decorin) were also evaluated. Among all tendon matrix‐related genes analyzed, collagen type I and tenascin showed no significant difference in the expression levels (Figure 3A(iii–vi)). However, co‐culture with vascular cells downregulates the expression of decorin while upregulating the expression of collagen type III. The same trend is found for collagen III/collagen I expression ratios, although without showing statistically significant differences between the two groups (Figure S6, Supporting Information). The expression levels of these structural ECM constituents can serve as indicative clues of the development stage or disease phenotype in tendon tissue.^[^ ^15^ ^]^ Developing tendons are known to have higher COL‐III levels, which are gradually replaced by the more mechanically competent COL‐I as the tissue matures.^[^ ^15^ ^]^ Similarly, damaged tendons also show increased COL‐III content that is gradually replaced by COL‐I over the remodeling phase of tissue repair.^[^ ^7^ ^]^ On the other hand, DCN is a vital player in the regulation of multiple biological processes, including fibrillogenesis. Its expression level is higher in aged/mature tendons,^[^ ^76^, ^77^ ^]^ where it plays important roles in maintaining tissue homeostasis,^[^ ^78^ ^]^ while its decreased content has been associated with a predisposition to tendinopathy.^[^ ^79^ ^]^ Interestingly, Thorpe *et.al* have recently identified a new specialized CD146^+^ cell population and vascular niche in the interfascicular matrix that expands following an injury.^[^ ^80^ ^]^ In contrast to what has been the traditional knowledge in the field, their findings support the growing evidence on the important role of vascular cell populations not only for progress of tendon pathologies but also in tendon development, homeostasis, and repair.

Having demonstrated the impact of the compartmentalized crosstalk between tendon core and vascular cells on hTDCs phenotype, we next evaluated its impact on the expression of several proinflammatory markers involved in the recruitment and activation of immune cells at inflammation sites. Figure 3C shows that the gene expression of some proinflammatory markers, including interleukin‐6 (IL‐6), interleukin‐8 (IL‐8), and chemokine ligand 2 (CCL2), is significantly elevated in the co‐culture system. Similar effects have been previously reported for co‐culture of renal fibroblasts with vascular cells.^[^ ^81^ ^]^ Interestingly, treatment of tenocytes with recombinant IL‐33, a proinflammatory cytokine expressed in endothelial cells and fibroblasts and whose increased expression is a characteristic of early tendinopathy, has also shown to induce the upregulation these proinflammatory mediators, as well as of COL‐III.^[^ ^82^ ^]^ Several studies have demonstrated that IL‐6, IL‐8, and CCL2 enhance the survival and migration of T cells,^[^ ^2^, ^83^, ^84^, ^85^ ^]^ highlighting the impact of multiple cell types, including vascular cells,^[^ ^86^ ^]^ in the progression or resolution of inflammation, supporting its relevance for accurately modeling these pathological conditions. These insights demonstrate that a deeper understanding of the crosstalk between tendon stroma crosstalk and the vascular system is required to decode its role not only in chronic tendinopathy, but also in tissue function,^[^ ^40^ ^]^ and should therefore be addressed in detail in future studies.

Overall, these results show that the combined biophysical and biochemical cues of the PL/sMRFs hydrogel matrix in the tendon core compartment synergistically support hTDCs tenogenic phenotype. When co‐cultured with endothelialized vascular‐like channels, hTDCs in the core show upregulation of TNMD and COL‐III and downregulation of DCN, suggesting that the multicellular crosstalk established in this microphysiological system guides hTDCs toward a more reparative‐like phenotype, evidence also supported by their more active proliferative profile and the increased expression of inflammatory markers. These features demonstrate that the proposed human 3D‐TenOC recreates key cell patterns and architectural hallmarks of tendon niche, providing an organotypic testing platform with functional advantages over traditional in vitro systems for modeling the mechanisms of tendon function and repair.

### 2.3 Adaptive Immune Responses of Inflamed Tendon Microenvironments are Modeled on 3D‐TenOC

#### 2.3.1 Extravasation and Migration Patterns of T Cells are Controlled by the Cellular and Chemical Environment

The role of cells from adaptive immune system, particularly T cells, in regulating the inflammatory response of other circulating immune cells and in activating resident tendon cells after injury is starting to be uncovered.^[^ ^19^ ^]^ However, very few mechanistic studies have directly screened T cell responses and their feedback on tendon tissues under relevant (patho)physiological conditions. Therefore, here, the onset of tendon tissue inflammation was selected as scenario to test the potential of the 3D‐TenOC as a platform for studying the response and effects of circulating T cells in tendon core compartment under relevant multicellular microenvironments.

For this, the core compartment of the 3D‐TenOC was stimulated with the proinflammatory cytokine IL‐1β on day 7 of co‐culture, inducing an inflamed state (Inf). The well‐known and easy‐to‐handle Jurkat T cell line was used as CD4^+^ cellular model.^[^ ^87^ ^]^ After inflammatory priming of the core compartment, on day 8, CD3/CD28 activated T cells (ATc) were introduced into the vascular channel in order to recreate the adaptive immune responses occurring under inflamed (Inf‐ATc) conditions, where T cells have been activated in lymph nodes by antigen‐presenting cells. The impact of non‐activated T cells (Tc) and ATc on tendon core without inflammatory treatment (C1‐Tc and C2‐ATc) were used as control groups.

**Figure 4:**
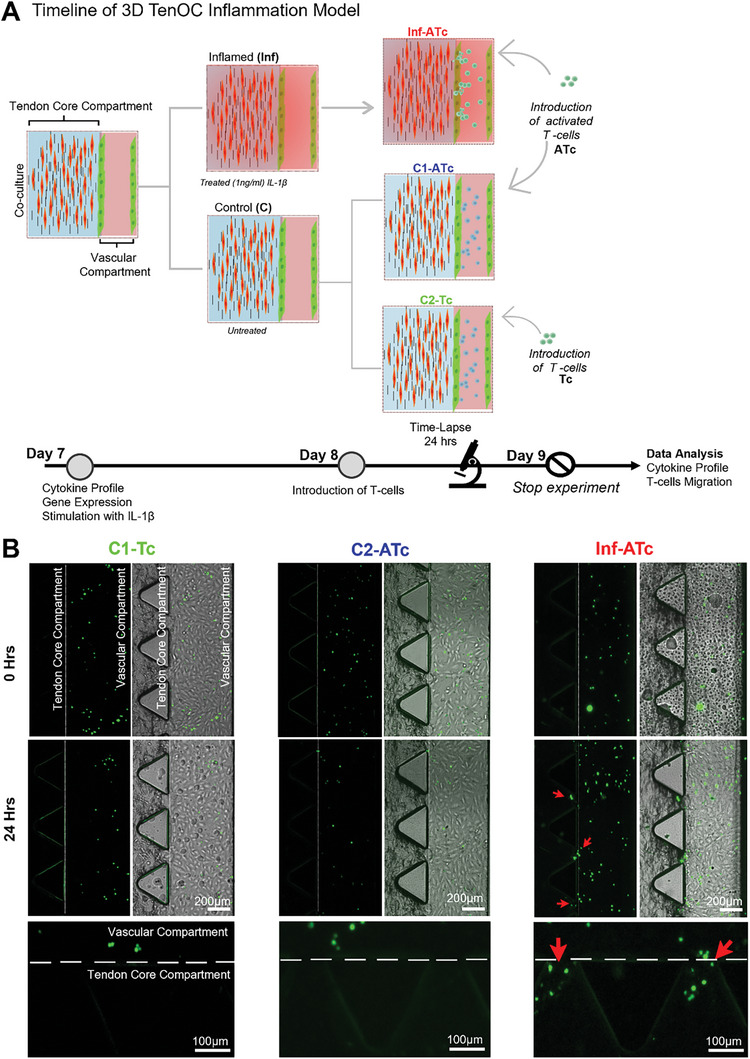
Inflammation 3D‐TenOC model. A) Overview of 3D‐TenOC inflammation model timeline. On day 7 of co‐culture, the microfluidic chips are separated into two groups. The first group consists of hTDCs that are untreated: control C), while the second group is treated with IL‐1β for 4 h inflamed (Inf). After this treatment, the chips are washed and kept in fresh media for 24 h. On day 8 of culture, T cells are introduced through the vascular channel. The C group is further divided into two subgroups. In one subgroup, non‐activated T cells (Tc) are introduced (C1‐Tc), while in the other subgroup, activated T cells (ATc) are introduced (C2‐ATc). To simulate crosstalk between cells during inflammation in (Inf), activated T cells are introduced (Inf‐ATc). Time‐lapse analysis is performed for 24 h, and on day 9 the supernatant is collected to evaluate the soluble cytokine levels and quantify the individual cell migratory data. B) The time‐lapse microscopy images are presented in two channels: fluorescent (left) and merged fluorescent with brightfield (right). The T cells are labeled with a green cell tracker. Scale bar: 200 µm. After 24 h (bottom), there is no extravasation of T cells in (C1‐Tc) and (C2‐ATc) groups, while in (Inf‐ATc) the extravasation of activated T cells can be observed with the majority of cells localized at the intersection between tendon core and vascular compartments highlighted by red arrows. On bottom magnified image is illustrated to aid in the visibility of T cell extravasation, Scale bar: 100 µm.

Prior to T cell loading in the vascular compartment, the barrier function of vessel structures was evaluated using fluorescent 20 kDa rhodamine‐dextran as a model diffusing molecule. In contrast to monoculture, chips with vascular endothelium retained more effectively the fluorescent dye within the channel (Figure S7,Supporting Information); however, as anticipated and in good agreement with previous studies,^[^ ^88^ ^]^ the apparent permeability of the endothelium in the inflamed model was significantly higher than the non‐inflamed model. T cell behavior was then followed in real‐time by fluorescence microscopy to monitor their transendothelial migration across the endothelial vessel (extrinsic compartment) into the adjacent tendon core compartment. Imaging analysis showed that a significant number of ATc were recruited toward the intrinsic compartment upon adherence to endothelial cells and subsequent extravasation (**Figure** **4**B; Figures S8–S10, Supporting Information). The quantification of fluorescently‐labeled T cells that extravasated into the intrinsic compartment showed significantly higher levels in the inflamed model (p = 0.0006) compared to the untreated groups, while the activation of T cells alone did not have a significant impact on their extravasation behavior (Figure 4B; Figure S8, Supporting Information). These observations are consistent with in vivo tendinopathic or tendon injury scenarios, where circulating immune cells are recruited at the site of injury via release of chemical mediators (e.g., cytokines and chemokines) by inflamed resident cells.^[^ ^8^, ^89^ ^]^ Besides stromal cells, inflamed endothelial cells most likely also contribute to these effects, considering that IL‐1β is known to induce the secretion of CXCL9 by endothelial cells,^[^ ^90^ ^]^ which is involved in facilitating the recruitment of effector T cells to the site of inflammation.^[^ ^91^ ^]^ Our results also show that, despite modulating the inflammatory profile of stromal cells, the chemotactic gradients generated from the co‐culture of hTDCs with vascular cells are not enough by themselves to induce significant recruitment of T cells into the core compartment.

It is important to highlight that, although transwell systems have been widely applied for cell migration studies, the presence of artificial membrane barriers, effects of gravity, and/or the inability to fully replicate natural physiological gradients can limit not only real‐time assessment options, but also the biological relevance of obtained results.^[^ ^92^, ^93^ ^]^ In contrast, our microphysiological system allows the observation of changes over time under representative multicellular scenarios and chemotactic gradient, which impact the complex cellular mechanisms involved.

**Figure 5:**
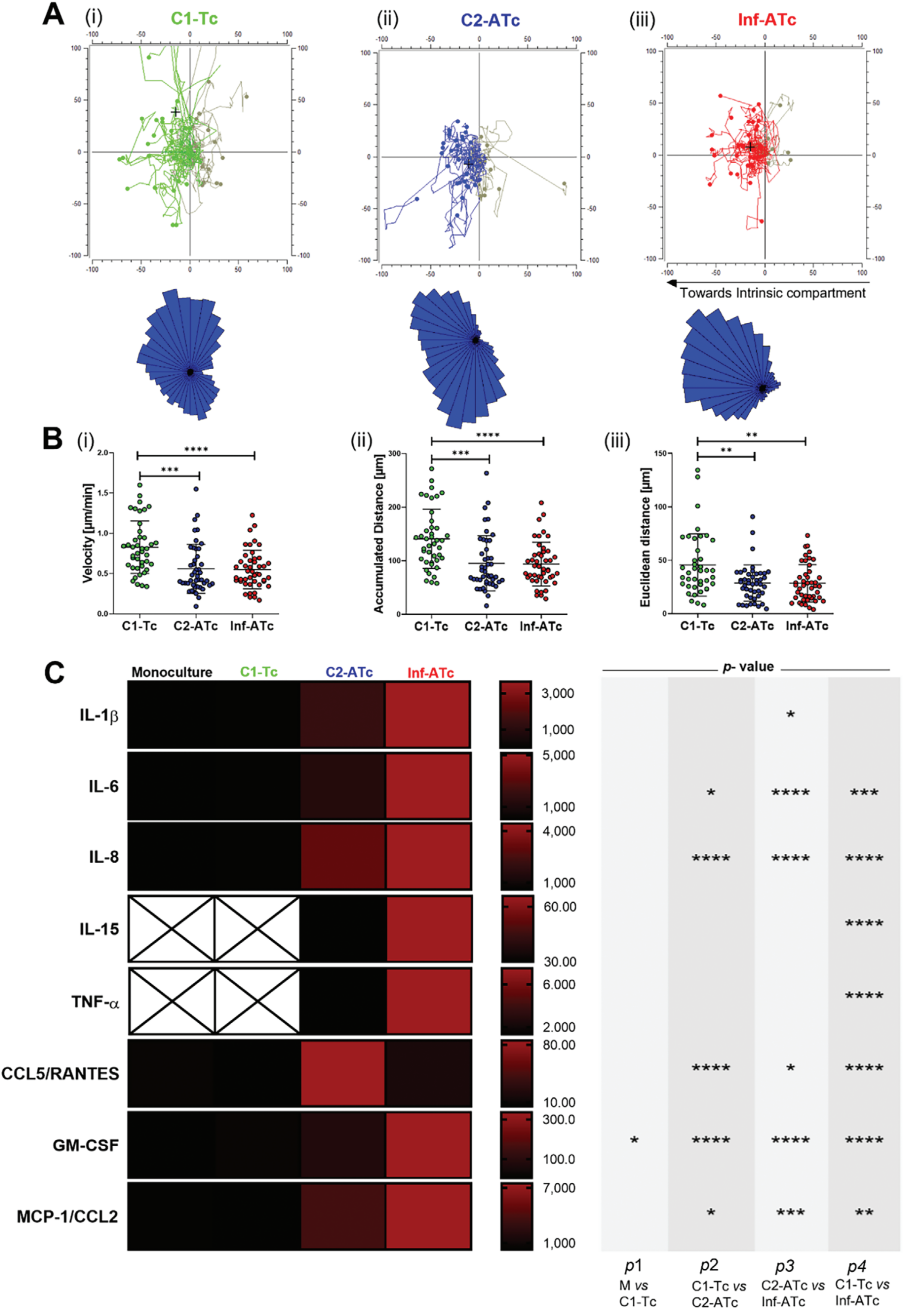
A) Individual cell trajectories of migrating T cells when co‐cultured with hTDCs. The line across the *x*‐axis (0, *y*) splits the circular positions to the left and right with 180° allocated to each direction. Colored lines highlight directions adopted towards the intrinsic compartment (left), while grey lines indicate movements in the direction away from the intrinsic compartment (right). The circular dots (i) control (C1‐Tc), (ii) control (C2‐ATc), and (iii) inflamed (Inf‐ATc) represent the endpoint location of individual cell paths. The black cross represents the center of mass at the end of the tracking. Rose plots illustrate the distribution of the trajectories at distinct angle intervals. Further extension of segments away from the center represents a larger proportion of cells with endpoints within the defined angle. B) Migration parameters quantified from cell trajectories toward the core compartment (i) velocity (µm/min), (ii) accumulated distance (µm), and (iii) Euclidean distance (µm), data presented as mean ± standard deviation (n = 45), statistical difference *p < 0.05 determined by one‐way ANOVA followed by Bartlett's test. C) Multiplex cytokine analysis of supernatant was performed. Cytokine levels were measured, and data are presented in pg mL^−1^. The supernatant was collected on day 9 immediately after the end of the time‐lapse experiment. Statistical difference was determined and represented as *p*1 for monoculture versus C1‐Tc, *p*2 for C1‐Tc versus C2‐ATc, *p*3 for C2‐ATc versus Inf‐ATc, and *p*4 for C1‐Tc versus Inf‐ATc. Data presented as mean ± standard deviation (n = 2), statistical differences *p < 0.05, ***p < 0.005, ****p < 0.0005 were determined by one‐way ANOVA followed by Brown‐Forsythe test.

Time‐lapse microscopy was used to follow T cell migration in real‐time (**Figure** **5**A and Movies [Link], [Link], [Link], Supporting Information). Cell migration trajectories showed that the majority of the T cells in inflamed conditions (Inf‐ATc) are significantly more attracted toward the core compartment in comparison to the control (C1‐Tc). The Rayleigh test was used to analyze the vector data of T cell migration in both conditions. Although the resulting *p*‐value (*p* = 0.025) for C1‐Tc indicates a slight clustering of T cell migration angles, its dispersion is not significantly different from a random distribution. On the other hand, the value (*p* = 0.000003) for Inf‐ATc indicates a much stronger clustering of T cell migration angles, indicating that there is a significantly preferred direction of migration. This difference in Rayleigh's values indicates that chemotactic gradients in the inflammation model have a remarkable effect on the direction of T cell migration towards the core compartment. The localization and activity of T cells over time can be used as indicator of the communication established both among themselves and with their surrounding environment (Figure S9 and Movies [Link], [Link], [Link], Supporting Information), which involves the information transfer through chemical signals or molecular interactions.^[^ ^94^ ^]^ T cells signaling is essential for coordinating the immune response and regulating T cell function.^[^ ^95^ ^]^ We observed that cells displayed a net motion towards the intrinsic compartment in both C1‐Tc and Inf‐ATc models. The tracking data obtained from these 3D‐TenOC models were analyzed to establish the differences in cell migration dynamics^[^ ^96^ ^]^ among the groups. The performed analysis of velocity, accumulated distance, and Euclidean distance showed notable differences between the control C1‐Tc and Inf‐ATc models. As expected, the non‐activated T cells in the C1‐Tc model covered a greater distance and moved at a faster pace compared to the activated T cells in the Inf‐ATc model (Figure 5B).

#### 2.3.2 Multicellular Crosstalk Impacts Soluble Inflammatory Secretome

To further evaluate how the crosstalk established between different cell populations under different inflammatory conditions affects the secretome of this microphysiological system, we next analyzed the profile of soluble inflammatory cytokine/chemokine mediators in the culture media on day 9 (Figure 5C). Tendon core monoculture was also included in these studies as a control group. Our aim was to investigate whether the inflamed microenvironment established after tendon tissue damage can or not be amplified by T cell response, and how core‐vascular interactions affect this mechanism. Several proinflammatory cytokines and chemokines with known roles in the recruitment and migration of immune cells,^[^ ^22^, ^97^, ^98^ ^]^ including interleukins (IL‐1β, IL‐6, IL‐8), regulated on activation, normal T cell expressed and secreted (RANTES) cytokine, monocyte chemoattractant protein‐1 (MCP‐1), and granulocyte‐macrophage colony‐stimulating factor (GM‐CSF), were detected in both mono and non‐activated co‐culture systems without showing major differences on their concentration levels. The interaction of tendon core with activated T cells promoted on its own a general increase in proinflammatory cytokine concentrations, which were markedly increased following inflammatory priming of the system. This is particularly evident on the levels of IL‐1β, IL‐8, and CCL2, but also on TNF‐α and IL‐15 expression, which were not detected in the mono and non‐activated co‐culture systems. Activation of tenocytes with TNF‐α is known to induce a further upregulation of TNF‐α, which subsequently plays a critical role in the regulation of matrix‐degrading enzymes and inflammatory cytokines,^[^ ^83^ ^]^ including the increase expression of IL‐6.^[^ ^99^ ^]^ CCL2 for example, is well known for its macrophage‐activating effects, but also as a T cells chemoattractant.^[^ ^100^ ^]^ The immunomodulatory consequences that can derive from our results are complex, but it tends to suggest that the crosstalk with vascular systems and circulating T cells does not have per se significant impact on the proinflammatory profile of the analyzed secretome. However, interaction with activated T cells is sufficient to induce a proinflammatory response. Interestingly, RANTES results behave differently from all other cytokines and chemokines analyzed. The detected levels of soluble RANTES, a potent CD4^+^ T cell chemoattractant,^[^ ^101^ ^]^ are lower in the inflamed tendon than under non‐stimulated conditions. We hypothesize that this effect may be related to the fact of inflamed cells expressing increased amounts of CCR5 receptors compared to the non‐inflamed cells,^[^ ^90^ ^]^ that in turn would lead to increased RANTES binding and immobilization on cell surface and reduce its soluble form levels. However, this hypothesis remains to be confirmed and should therefore be further evaluated in future studies.

Overall, our results are consistent with earlier findings at the gene expression level derived from direct co‐cultures of tenocytes with T cells,^[^ ^22^ ^]^ supporting the existence of proinflammatory feedback loops between T cells and tenocytes, established not only among tissue‐resident cells but also with cell recruited from the circulatory system that can amplify inflammation and contribute for disease progression, leading to chronicity. Importantly, the results of core compartment phenotype characterization and cell tracking data, suggest that this proinflammatory vicious cycle seems to be potentiated by the presence of vascular vessels in the vicinity of tendon core, which is known to be increased in tendinopathy.

In summary, our human 3D‐TenOC model allows the accurate replication of different scenarios of the tendon microenvironment observed on the onset of tendon inflammation preceding tendinopathy, making it a potential platform for investigating the relationship between multiple cell types involved in tendon pathophysiology. Our results confirm earlier findings on the importance of the interaction between the tendon core compartment and adaptive immune system on the amplification of tendon inflammatory responses,^[^ ^22^ ^]^ and further allowed to study for the first time in vitro the mechanisms of recruitment and extravasation of T cells from the vascular system under controlled conditions. The faithful recreation of tendon niche on a microfluidic chip demonstrates that our platform can be used as a relevant 3D tendon in vitro model, enabling further mechanistic research on tendon tissue physiology and pathophysiology mechanisms. Considering the magnetic responsiveness of the sMRFs incorporated in the core compartment in our design, one particular avenue to be explored is the evaluation of the remote stimulation capability of magnetic biomaterials as a strategy for resolving inflammation and promoting tendon regeneration under a relevant multicellular microenvironment.

Our 3D‐TenOC has limitations. For practical reasons, endothelial and T cell lines were used under this study. However, cell lines behave differently than primary cells, which should therefore be considered in future studies exploring this modeling platform. Additionally, further characterization of specific T cell subtypes present in the model would provide a more precise understanding of the immune responses involved in tendon inflammation. The timeframe of our experiments is well adapted to assess the early cell behavior occurring at the onset of inflammatory process. However, to comprehensively understand cellular events, including tendon stroma‐vascular crosstalk, further investigation is required for a more comprehensive understanding of cellular cross talk effects. Nevertheless, studies focused on cellular events and crosstalk happening in established tendinopathy will require additional testing groups recreating, e.g., the hallmarks of fibrotic tendon tissue occurring under those conditions. In that sense, some fabrication potentialities of our system, such as the ability to control cell organization within the microfluid device by controlling sMRFs orientation with magnetic fields, are features that can be leveraged on the design of those microphysiological systems. Moreover, although IL‐1β has been traditionally used as the main inflammatory inducer in models of inflammation, single cytokine do not fully represent the biological complexity of in vivo inflammatory conditions.^[^ ^2^, ^22^ ^]^ Therefore, additional cytokines playing key roles in tendon inflammation process (e.g., TNF‐α) should be consider to enhance the physiological representative of the model.^[^ ^69^, ^89^ ^]^ Like in most microfluid devices, the retrieval of cellular samples from micron scale channels/chambers is challenging, limiting the downstream biological analysis that can be performed. Although miniaturization is a key feature of organ‐on‐chip, the dimension and device design should be improved the mitigate these limitations and expand the conclusion that can be drawn from future generations these as models. Finaly, it worth mentioning that although our results have been discussed based on exiting literature from both animal and human data, further validation with clinical observations from human pathology specimens that inform our model will contribute to increase the impact of this platform.

## 3 Conclusion

In this study, we combined magnetic materials, bioactive hydrogel matrices, and different tendon cell populations to bioengineer a 3D‐TenOC recreating the intricate ECM architecture and cellular patterns of tendon tissue. We devised a new strategy capable of guiding cell alignment on the chip by magnetic manipulation of sMRFs within xeno‐free and bioactive PL hydrogel matrices. This approach revealed effective on synergistically driving the development of a tenogenic phenotype in the core compartment, which could be interfaced with perfusable vascular channels to accurately represent in vitro tendons’ intrinsic and extrinsic compartments. The phenotype of the bioengineered core resulting from the multicellular crosstalk in this microphysiological system is consistent with a tendon repair profile. This 3D‐TenOC was herein used to assess for the first time the immunomodulatory feedback of multicellular communication during the onset of tissue inflammation, focusing on adaptive immune cell responses. We have shown that T cells arrest, extravasation, and migration mechanism typically seen in vivo are recreated in our 3D‐TenOC, and further confirmed that T cells have a proinflammatory effect over tendon core compartment, contributing for supporting the growing evidence on their key role on the mechanisms leading to chronic tendinopathy. Overall, the developed 3D‐TenOC platform can be a valuable tool for investigating the spatio‐temporal dynamics of the cellular and molecular mechanisms driving the pathogenesis of tendinopathy and for testing new therapeutic strategies for this debilitating disease.

## 4 Experimental Section

### Zn‐MNPs and sMRFs Synthesis

Spherical iron oxide‐based MNPs doped with zinc with 9.0 ± 0.8 nm in diameter were prepared through thermal decomposition processes according to a previously optimized procedure.^[^ ^43^ ^]^ Briefly, 2.4 mmol of iron (III) acetylcetonate, 0.6 mmol of zinc (II) acetylacetonate, 10 mmol of 1,2‐hexadecanediol, 6 mmol of oleic acid, 6 mmol of oleylamine and 20 mL of benzyl ether were added to the flask (all reagents purchased from Sigma‐Aldrich). The temperature of the mixture was increased to 110 °C and then a 10 mbar vacuum was applied for 1 h under continuous magnetic stirring. After that, the vacuum was exchanged by nitrogen flow and the temperature was increased to 210 °C for 2 h (heating ramp 8 °C·min^−1^) and finally to 295 °C for 1 h (5 °C·min^−1^). After cooling down to room temperature, an excess of ethanol was added to the solution before precipitating the Zn‐MNPs with a magnet.

PCL‐based sMRFs were prepared through electrospinning. PCL (Mn = 80 000, Sigma‐Aldrich) was dissolved at 17% (w/v) concentration in a 70/30 (v/v) chloroform/N,N‐dimethylformamide (DMF) mixture (from Laborspirit and Fisher Scientific, respectively). Prior to mixing the solvents, Zn‐MNPs were dissolved in the chloroform phase (5% (w/w) respect to PCL), exploiting their high stability in this solvent provided by their hydrophobic surface coating. The solutions were magnetically stirred for 24 h and electrospun at flow rate and voltage conditions of 1 mL·h^−1^ and 13.1 kV, respectively, being the distance between the tip and the collector being 12.5 cm. After that, the electrospun membranes were embedded in OCT (Sigma‐Aldrich) blocks that then were frozen at ‐20 °C and microcut at 25 µm thickness using a cryostat microtome. The obtained short microfibers were washed several times with water to remove the OCT and finally redispersed with ultrasounds.

### Magnetic Field Generation Setups

For the magnetic setup, a poly‐lactic acid (PLA, Mitsubishi Chemicals Performance Polymers, USA) mold was 3D printed in a B2×300 3D printer (Beeverycreative, Portugal) after preparing a CAD design with the dimensions required to place three pairs of parallel neodymium magnets 2.5 cm apart, creating 85–100 mT magnetic field. For this study, commercially available microfluidic chips were used (AIM DAX‐1 3‐D cell culture chip, AIM biotech, Singapore). The microfluidic chips were placed in the custom‐made 3D printed mold to hold the magnets to enable the alignment of encapsulated sMRFs (see section Activation of T Cells below) under the influence of the magnetic field. The computational simulation of the magnetic field generated by the parallel magnets setup was performed using COMSOL Multiphysics Software, v. 5.4.

### Isolation and Culture of Human Tendon‐Derived Cells (hTDCs)

hTDCs were isolated from surplus healthy tissue samples of patellar tendons, and collected from adult patients undergoing orthopedic reconstructive surgeries under protocols previously established with Hospital da Prelada (Porto, Portugal) and with informed consent of the patients. The content of the written informed consent and related procedures were reviewed and approved by the Hospital Ethics Committee (P.I. N.°005/2019). The tendon tissue identification and quality (healthy/diseased) were assessed by the medical team during surgical intervention.

Following a previously established protocol,^[^ ^102^ ^]^ tendon samples were minced using a sterile scalpel. Phosphate buffer saline (PBS) drops were added continuously to keep a moist environment and reduce cell damage by mechanical forces. The excess PBS was removed using a filtration system for 50 ml tubes (Falcon). Minced samples were collected into a 50 mL tube, already containing an enzymatic solution of collagenase (0.1%, Sigma‐Aldrich, C6885, USA) with 2 M CaCl2 (1:1000, VWR, Germany) and 1% bovine serum albumin (BSA) (Sigma–Aldrich, USA) for 1 h at 37 °C under constant agitation. A ratio of 1:1 of minced tissue to the enzymatic solution was considered. After incubation, digested samples were filtered and centrifuged three times at 290 g for 5 min, and the supernatant was discarded. Isolated hTDCs were expanded in α‐MEM medium (A‐MEM, Invitrogen, Life Technologies Limited, Paisley, UK) composed of α‐MEM supplemented with 10% fetal bovine serum (FBS) (Alfagene, Life Technologies Limited, UK) and 1% antibiotic/antimycotic solution (A/A) (Alfagene, Life Technologies Limited, UK) in humidified 5% CO_2_ atmosphere. hTDCs from passages 1 to 3 were used to perform all the experiments.

### Cell Culture of Vascular and T Cells

The endothelial cell line EA.hy926 (ATCC CRL‐2922) was commercially obtained from ATCC (LGC Standards, UK) and maintained in culture using low glucose Dulbecco's modified Eagle medium (DMEM, Gibco) supplemented with 10% FBS (Thermo Fisher Scientific) and 1% antibiotic/antimycotic solution (Sigma–Aldrich). For T cells, Jurkat Cell Line expressing tGFP (P20128‐GVO‐IP) was commercially obtained from BioCat GmbH, Germany, and maintained in culture using advanced RPMI 1640 (Gibco/Invitrogen) with 10 mM Hepes, supplemented with 10% FBS (Thermo Fisher Scientific) and 1% antibiotic/antimycotic solution (Sigma‐Aldrich).

### Activation of T Cells

For T cell activation, Dynabeads Human T‐Activator CD3/CD28 (Thermo Fisher Scientific) was used according to manufacturer's instructions. Briefly Jurkat cells (BioCat GmbH, Germany) were cultured in 6‐well plates (Costar, 734–1599) in Advanced RPMI 1640 (Gibco/Invitrogen) with or without CD3/CD28 Human T‐activator Dynabeads (Thermo Fisher Scientific) at a bead‐to‐T‐cell ratio of 1:10 for 48 h, resulting in stimulated and unstimulated T cells, respectively. The stimulated cells were separated with DynaMag (Thermo Fisher Scientific) and used for further experiments.

### Fabrication of 3D‐TenOC

The microfluidic chip (AIM DAX‐1 3‐D cell culture chip, AIM biotech, Singapore) was placed in the custom‐made magnetic system as described above. For seeding hTDCs (2.10^6^ cells mL^−1^) in the central compartment of the microfluidic chip, frozen aliquots of PL were thawed at 37 °C for 5 min, centrifuged at 4000 G for 5 min (Centrifuge 5810 R, Eppendorf) and then filtered with a 0.45 µm pore membrane filter prior to being mixed with the trypsinized cell pellets. After that, sMRFs (2 mg·mL^−1^) were added along with the thrombin (5 U·mL^−1^) and calcium chloride (100 mM). 10 µL of this mixture was loaded into the central chamber of the chip (6 µL from the top inlet and 4 µL from the bottom inlet) without forming a bubble and the chip was then left at rest under the uniform magnetic field. Similar experiments without cells were followed during the optimization of the fabrication steps. Following gel formation (occurring about 10 min after loading) when the alignment of sMRFs was locked, vascular cells (6.10^6^ cells mL^−1^) were introduced from the lateral channel. The chip was flipped twice within 30 min to ensure adherence of cells to the lateral sides of the channel. After half an hour, media was circulated in the lateral channel to remove the non‐adhered cells, and the chip was maintained in an incubator for a week with fresh α‐MEM medium for monoculture and α‐MEM and DMEM media combined in equal parts (1:1) for coculture, in both cases supplemented with 10% FBS.

For modeling inflammation, hTDCs seeded in the central compartment of the chip were supplemented with 1 ng·mL^−1^ of IL‐1β^[^ ^103^ ^]^ (Alfagene, Life Technologies Limited, UK) in the cell culture media on Day 7 for 4 h, as previously established.^[^ ^22^ ^]^ Afterward, the cells were washed twice with PBS and kept for one day in culture with fresh α‐MEM and DMEM medium.

On day 9 of culture, the vascular channel was used to introduce the active or non‐activated T cells at a cell density of (5·10^5^ cells·mL^−1^). Fresh culture media containing equal parts of α‐MEM, DMEM, and RPMI was added every day to replace the old media.

### Fluorescence Staining of 3D Compartmentalized Model

For immunostaining, all the solutions were added from the side channels of the microfluidic chip. The cells were fixed with 4% paraformaldehyde (Thermo Fisher Scientific) at room temperature for 30 min. Then 0.2% Triton X‐100 in PBS was used to permeabilize the cell membrane for 1 h at room temperature under gentle agitation. Next, the samples were blocked with 3% (w/v) BSA in 0.2% (w/v) Triton X‐100 in PBS for 1 h. After washing the samples three times with PBS they were incubated with primary antibody against SCX (Abcam 1:200) or TNMD (Abcam 1:200) or Collagen I (Abcam 1:500) or Collagen III (Abcam 1:100) or Ki67 (Abcam 1:200) and for endothelial cells anti‐CD31(PECAM‐1)‐APC antibody (Thermo Fisher 1:200), diluted in 1% (w/v) BSA in 0.2% (w/v) Triton X‐100 in PBS at 4 °C overnight with gentle agitation. This was followed by incubation for an additional day at 4 °C with the respective AlexaFluor‐488 secondary antibodies. The cytoskeleton and nuclei were stained with DAPI and Phalloidin for 1 h (DAPI, Sigma‐Aldrich, 1:1000 dilution; Phalloidin conjugated with rhodamine, Sigma‐Aldrich, 1:200 dilution). Samples were analyzed by confocal laser scanning microscopy TCS SP8 (Leica Microsystems, Germany) and were shown as maximum projection of the obtained *z* stacks. (n = 3, independent experiments).

### mRNA Extraction and Real‐Time RT‐PCR

Total RNA was extracted from the constructs using Trizol extraction reagent (TRI Reagent‐T9424Sigma Life Science) according to the manufacturer's instructions. Briefly, the vascular cells in the side channel were eliminated through a process of trypsinization, followed by two washes with PBS. Besides the trypsinization procedures adopted to detach ECs within the channel, in order to mitigate the potential risk of cross contamination, material was exclusively recovered from 2/3rd of the central/core compartment to eliminate any potential contribution from the vascular compartment. This implied first marking the region to be excluded with a marker and then cut the adhered thin plastic of the chip bottom at the core with a scalpel to avoid dragging the cells and material within the posts that separate compartments when opening the chip by detaching the bottom. Subsequently, the chip was opened, and the sample from the central compartment was collected. The quantity and quality of extracted RNA were analyzed with a NanoDrop ND‐1000 spectrophotometer (NanoDrop, ThermoScientific, USA). The cDNA synthesis was performed by qScript cDNA SuperMix kit (Thermo Fisher Scientific). The quantitative polymerase chain reaction (qPCR) was carried out for the quantification of the transcripts using the PerfeCTASYBR Green FastMix kit following the manufacturer's protocol, in a Real‐Time Mastercycler Realplex thermocycler (Eppendorf, Germany). The primers were pre‐designed with Primer3 and BLAST (NCBI, Bethesda, MD, USA) (Supporting information Table 1). GAPDH (Glyceraldehyde 3‐phosphate dehydrogenase) was used as the reference gene. The Delta‐Delta Ct Method was selected to evaluate the relative expression level for each target gene. All values were first normalized against GAPDH values, and then to hTDCs collected on day 0. A total of 25 chips per condition (either monoculture or co‐culture) and per doner (2) were produced. For practical reasons, the cellular material retrieved from the chips of the two different donors in each experimental replicate were pooled in order to enable the extraction of enough mRNA to perform the RT‐PCR analysis. Three sets of independent experiments were performed (n = 3).

### Cytokine Profile with Luminex Assay

Culture media of all groups was collected from the side channels of microfluidic chips on day 9. A total of 10 chip produced for each doner (2) and experimental group were pooled to produce the experimental replicates of each tested condition. Two sets of independent experiments were performed (n = 2). The collected supernatant was centrifuged to remove suspended cells and stored at −20 °C until further analysis. The biological replicates were measured in triplicates. For multianalyte profiling human cytokine magnetic 25‐plex panel (Thermo Fisher Scientific, UK) was used according to the manufacturer's instructions to identify and measure the release of soluble cytokine/chemokines due to cellular crosstalk. The concentration of each analyte: interleukins (IL‐1β, IL‐6, IL‐8, IL‐15), tumor necrosis factor‐alpha (TNF‐α), C–C motif chemokine ligand 2 (CCL2), regulated on activation, normal T cell expressed and secreted (RANTES), monocyte chemoattractant protein‐1 (MCP‐1), and granulocyte‐macrophage colony‐stimulating factor GM‐CSF was calculated using the Luminex xPONENT 4.2 software and illustrated in pg/mL.

### Live Timelapse Microscopy and Cell Migration Analysis

Cell migration studies were conducted using an inverted epifluorescence microscope with environmental control (5% CO_2_ and 37 °C) (DMi 8, Leica). The chip was placed inside the microincubation chamber of the microscope on visualizing stage and 50 µL of T cells solution (20000 cells) was injected into the inlet/ followed by the addition of 50 µL medium to the outlets. Image acquisition was set at a frequency of 1 image each 15 min for 24 h. Acquired images were exported as a video file and imported into ImageJ (NIH) for analysis. Videos were first converted to 8bits to reduce rendering times. A manual tracking plug‐in for ImageJ was utilized to generate tracking data from each cell. For each group, 45 cells were selected at random within a fixed field of view. The obtained data were imported into the Chemotaxis and Migration tool V2.0 provided by ibidi. Cell migration trajectories were generated in relation to the paths adopted by the cells for each group along with shifts in the center of mass. Endpoint parameters were extracted in terms of velocity, accumulated distance, and Euclidean distance.

### Barrier Permeability Assay

The 20‐kDa dextran rhodamine (Thermo Fisher Scientific) molecule was used as a fluorescent tracer agent to assess the permeability across the endothelial barrier formed after seven days of cell culture. Briefly, the chip was placed inside the microincubation chamber of the microscope on visualizing stage (Inverted epifluorescence microscope with environmental control (5% CO_2_ and 37 °C) (DMi 8, Leica). The media in lateral channel was replaced by of 50 µL of 20‐kDa Dextran rhodamine solution (25 µM in cell medium). Fluorescence images were captured immediately after injection and for every 5 s for a total of 60 min. The diffusion of the dextran over time was analyzed by quantification of spatial changes in mean fluorescence intensity on the acquired images plotted against the distance from the vascular/lateral channel. ImageJ software was used to analyze the fluorescence intensity for measuring the diffusion from the lateral channel into the core compartment, in all three tested conditions: monoculture (hTDCs in core/central compartment), Coculture Control (vascular cells in lateral compartment and hTDCs in core compartment), and Coculture Inflamed (vascular cells in lateral compartment and hTDCs primmed with IL‐1β in core compartment). For analysis of the change in fluorescence intensity at 0 min and 60 mins, specific positions and areas were selected in the middle of the lateral compartment (vascular), at the interface between lateral and central compartment (interface), and within the central compartment (core). Data were mean ± s.d. Statistical differences between groups were determined by unpaired t‐test. Images were representative of 3–6 images per group. All data were plotted after fluorescence intensity normalization.

### Immunohistochemistry of Human Tendon Samples

Human tendon samples were obtained from cadaveric plantar flexor tendon (female, 56 years. The study was approved by the Balearic Islands Ethical Committee (IB4953/22 PI). Samples were formalin‐fixed and paraffin embedded. 5 µm sections were cut, deparaffinized and rehydrated. Sections were stained with alcian blue, eosin and hematoxylin, following standard protocols. For immunostaining, antigen retrieval was performed at 60 °C for 3 h in citrate buffer (10 mM sodium citrate tri‐hydrate pH 6.0, 0.05% Tween 20). Sections were blocked using 2% BSA in PBS‐Tween (0.05% Tween 20) for 1 h at room temperature. Anti‐Alpha smooth muscle actin mouse primary antibody (sc‐53142, Santa Cruz Biotechnology) was incubated overnight at 4 °C at 1:400 dilution in 2% BSA in PBS‐Tween. Sections were washed and incubated in fluorescent secondary antibody (Alexa Fluor 488‐conjugated Goat anti‐Mouse IgG H+L antibody, A‐11001, Invitrogen) at a concentration of 1 µg/mL in 0.5% BSA in PBS‐Tween for 1 h at room temperature. DAPI counterstaining for nuclei was performed for 30 min at room temperature kept in dark. Sections were mounted using aqueous mounting media (Fluoromount, F4680, Sigma‐Aldrich). Images were taken with a confocal microscope (LSM710 Carl Zeiss, Microcospy Platform, IdISBa). Image analysis and processing was performed using ImageJ software. (n = 1)

### Statistical Analysis

GraphPad Prism 8 software was used for statistical analysis. The reported values correspond to the means of a minimum of three independent experiments, unless otherwise specified. Data were presented as means ± SD.  Comparisons were performed using one‐way ANOVA followed by Bartlett's test for normally distributed or Brown‐Forsythe test for non‐normal data distribution. To compare two experimental groups unpaired two‐tailed Student's *t*‐tests were used. Values were considered significant when *p* < 0.05.

## Conflict of Interest

The authors declare no conflict of interest.

## Supporting information

Supporting Information

Supplemental Movie 1

Supplemental Movie 2

Supplemental Movie 3

Supplemental Movie 4

Supplemental Movie 5

Supplemental Movie 6

## Data Availability

The data that support the findings of this study are available in the supplementary material of this article.
