# Supplementary material for: Human Tendon‐on‐Chip: Unveiling the Effect of Core Compartment‐T Cell Spatiotemporal Crosstalk at the Onset of Tendon Inflammation
Source: Adv Sci (Weinh). 2024 Sep 11;11(41):2401170. doi: 10.1002/advs.202401170 (PMC11538684; doi:10.1002/advs.202401170)
Supplement: Supplementary file 1 — Supporting Information [file ADVS-11-2401170-s003.docx]

**Supporting Information**

**Human Tendon-on-Chip: Unveiling the Effect of Core Compartment-T Cell Spatiotemporal Crosstalk at the Onset of Tendon Inflammation**

Syeda M. Bakht, Alberto Pardo, Manuel Gomez-Florit, David Caballero, Subhas C. Kundu, Rui L. Reis, Rui. M. A. Domingues* and Manuela E. Gomes*


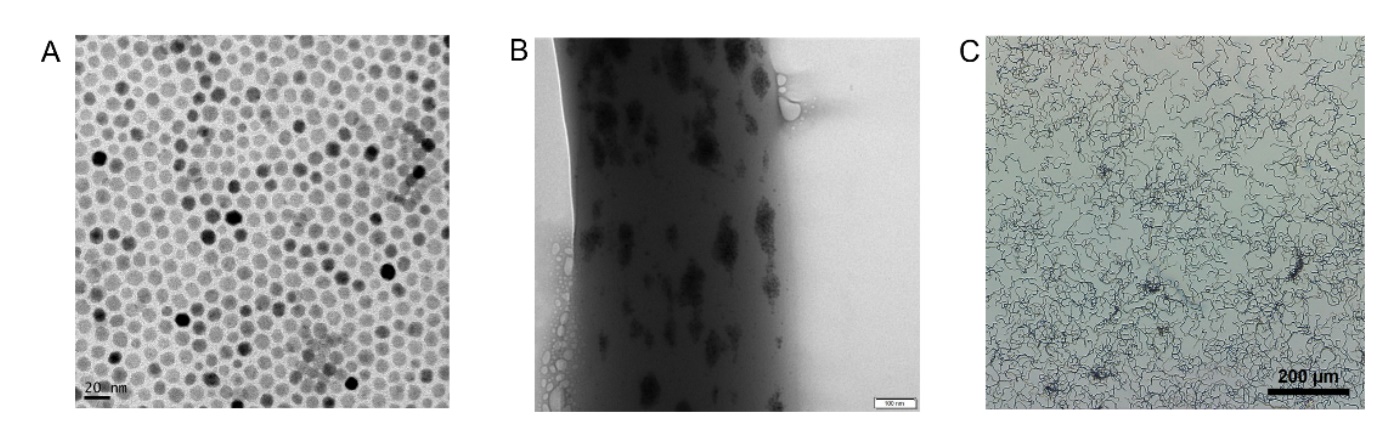


**Figure S1**: A) High magnification TEM image of Zinc-doped magnetic nanoparticles (Zn-MNPs) (scale bar 20 nm). B) TEM image of a PCL-based sMRFs incorporating 5% (w/w) MNPs (scale bar 100 nm). C) Optical microscopy images of cryosectioned short magnetic responsive fibers (sMRFs) with average length 41 ± 11 µm (scale bar 200 µm).


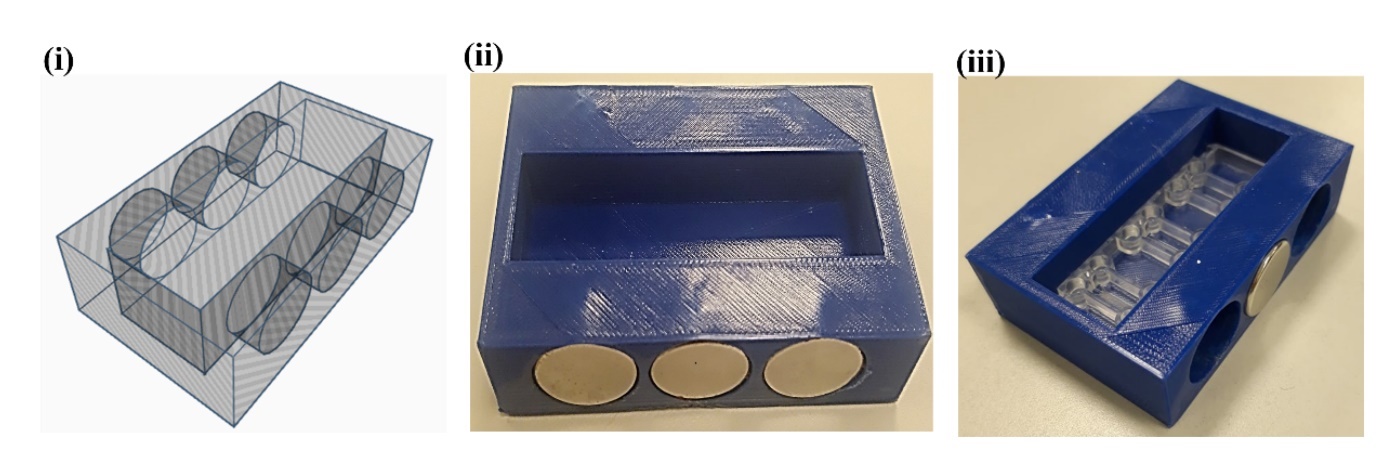


**Figure S2**: (i) CAD design of mold for alignment of magnets, (ii) magnets fixed and aligned within 3D printed PLA mold, (iii) commercial compartmentalized microfluidic inside the 3D printed mold.


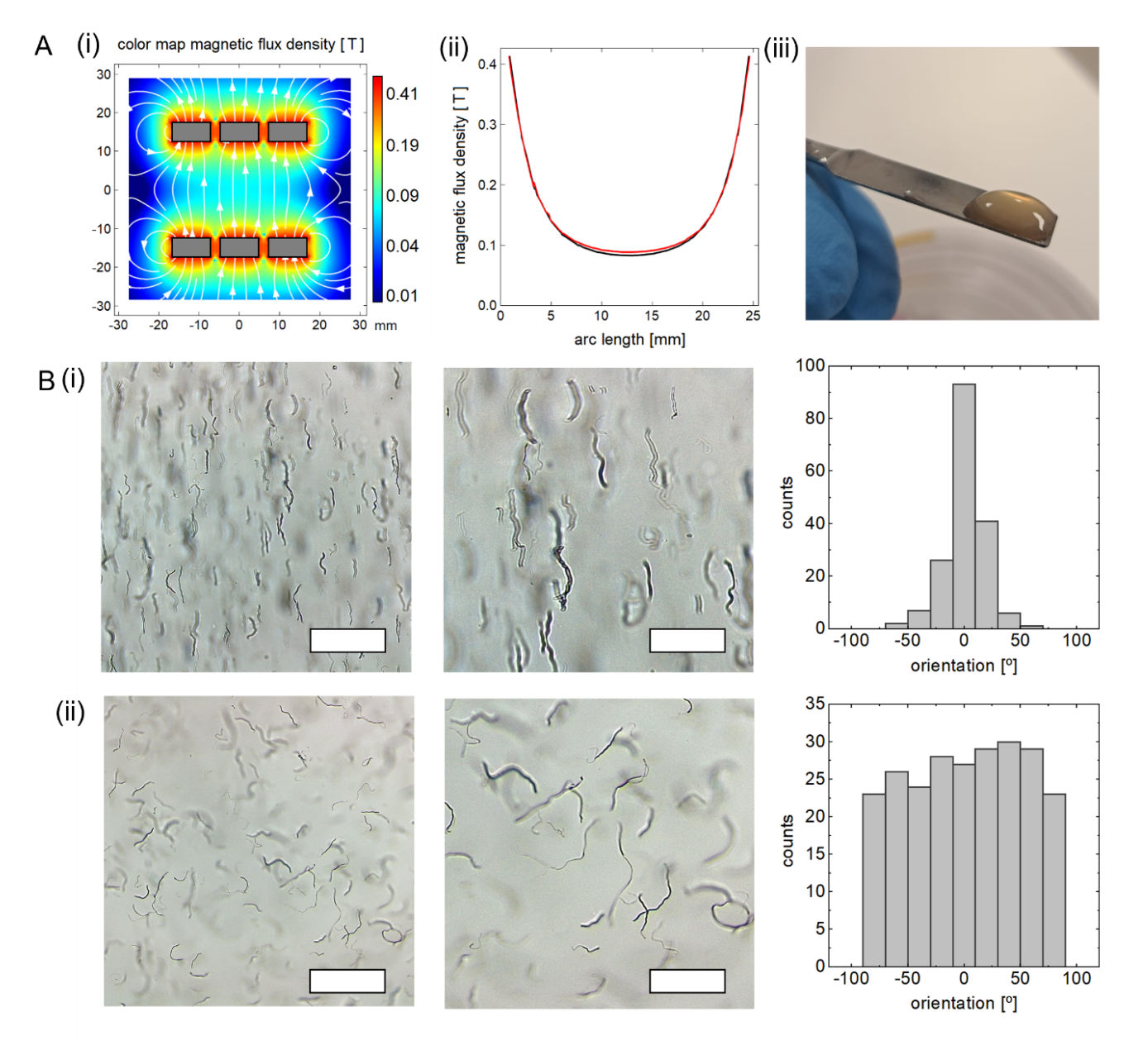


**Figure S3**: A) Computational simulations of the magnetostatic field created on the PLA mold; i) color map of magnetic flux density in the 2D plane connecting the centers of two parallel neodymium magnets (horizontal plane z = 1.05 cm; magnets height = 2.10 cm) and ii) simulation of the magnetic flux density along the 1D line between the centers of two neodymium magnets, iii) Gelation of platelet lysate (PL) with thrombin (5U mL^-1^) and calcium chloride (100 mM) with embedded sMRFs. B) Optical microscopy images of sMRFs aligned within PL and orientation statistical distributions of 41 ± 11 µm sMRFs modified with 5% (w/w) MNPs under i) 85 ± 15 mT applied magnetostatic field, and ii) no applied magnetic stimuli.


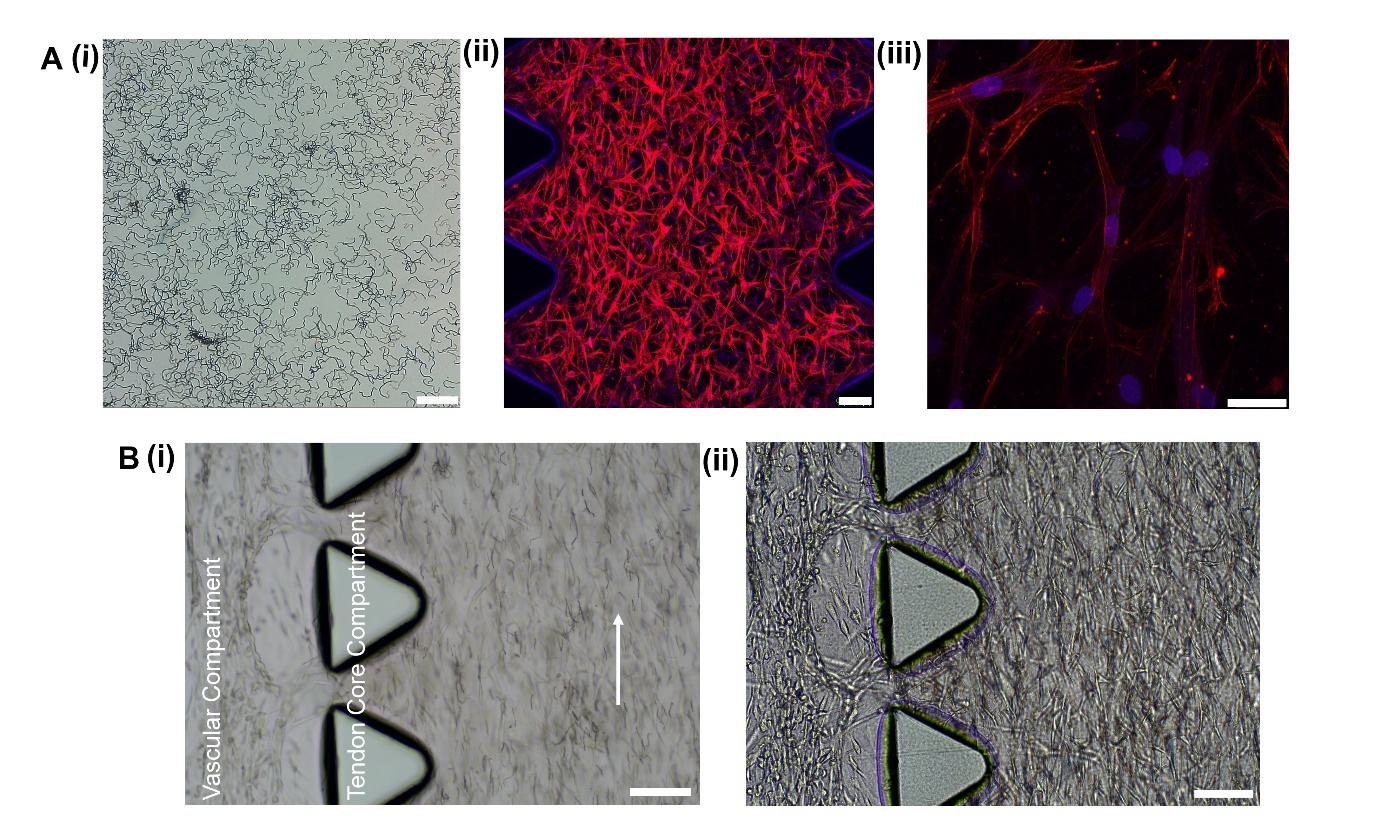


Figure S4: A) CLM images of sMRFs randomly embedded in platelet lysate hydrogel in absence of magnetic field i) Image of sMRF (fluorescent and brightfield merged channel), (ii) and (iii) Flurescent image for actin cytoskeleton (red) and nuclei (blue) of hTDCs embedded within platelet lysate hydrogel where cells follow the random orientation of sMRFs (scale bar: 100 µm, 100 µm, and 25 µm, respectively). B) Brightfield image of the coculture model on Day 7 in the central (intrinsic compartment) i) sMRF aligned in direction of applied magnetic field ii) hTDCs orient preferentially in the direction of sMRF, while vascular cells are compartmentalized in the side channel (vascular compartment) (scale bar: 200 µm). Red arrow indicates the sMRFs while the white arrow indicates the direction of the magnetic field.


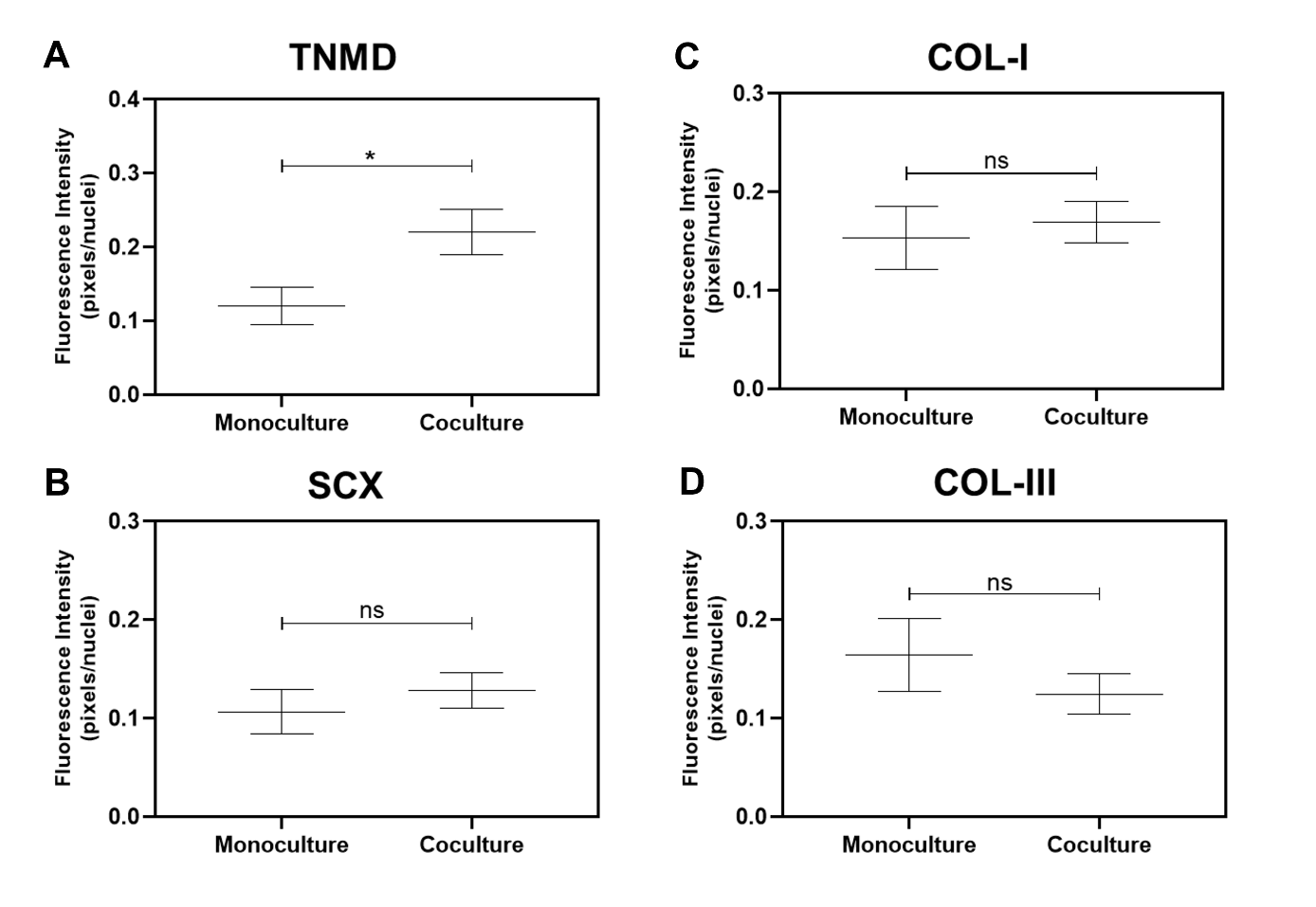


Figure S5: Protein expression of tendon-related markers in the core compartment of the 3D-TenOC. Phenotype markers (A) tenomodulin (TNMD) **p* < 0.05 and (B) scleraxis (SCX); and for matrix components, (C) collagen type 1 (COL-I), and (D) collagen type III (COL-III). Statistical significance was determined by an unpaired two-tailed t-test.


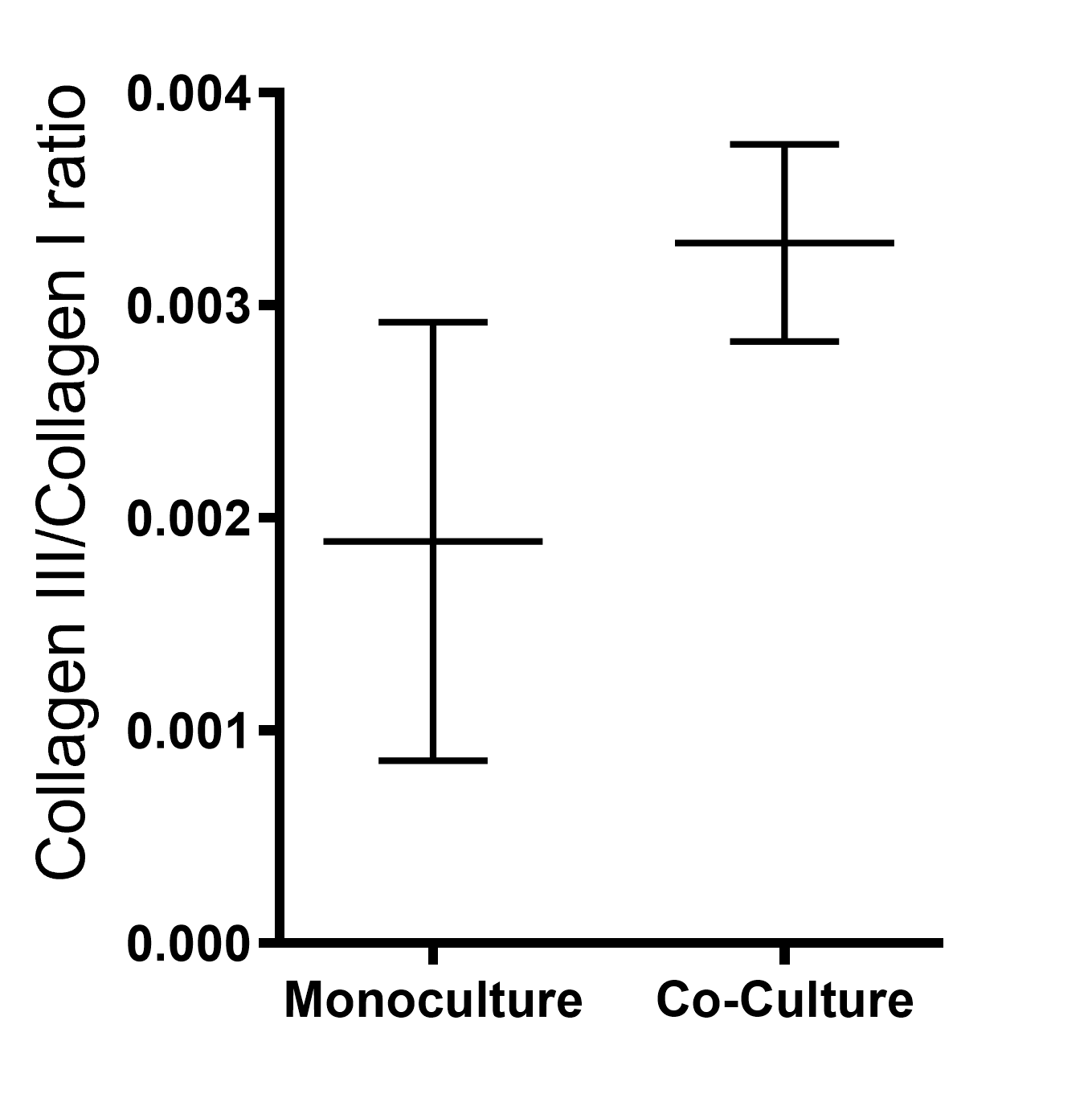


Figure S6: Ratio between relative expression of collagen III and collagen I.


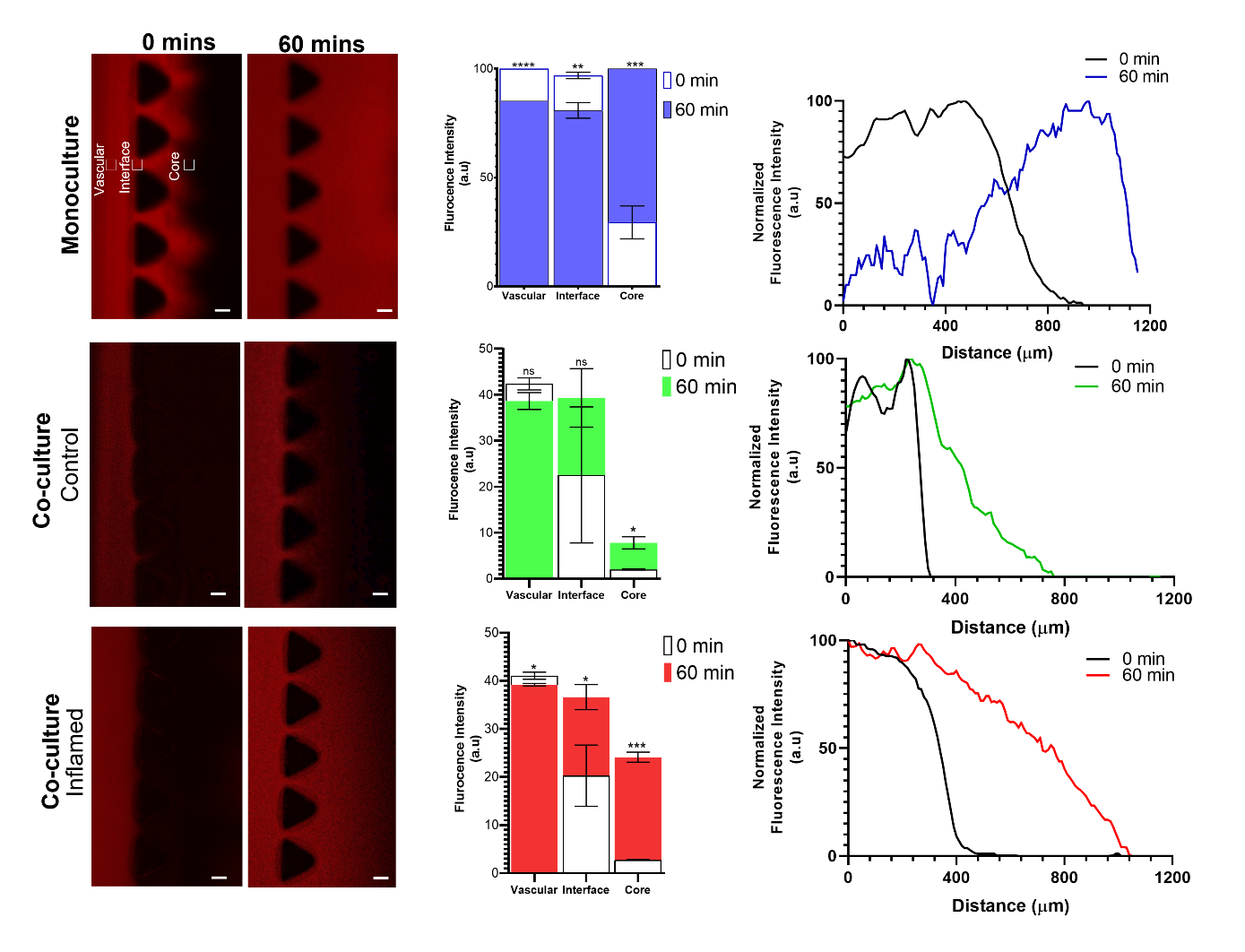


Figure S7: Time-lapse fluorescence images of a vascular channel filled with dextran 20 kDa at 0 min and at 60 min postinjection. Plot of normalized spatiotemporal fluorescence intensity for dextran 20 kDa, black dotted line represents 0 min while solid line blue, green and red lines represent detected fluorescence in monoculture, control and inflammed respectively (scale bar 100 µm). The bar plots represents the statistical diffrences between 0 min and 60 min at randomly selected points in lateral compartment (vascular), at the junction between lateral and central compartment (interface) and at the central compartment (core) as illustarted by square in image (monoculture) m. Data are mean ± s.d. Statistical differences between groups were determined by unpaired t-test. Images are representative of 3–6 images per group.


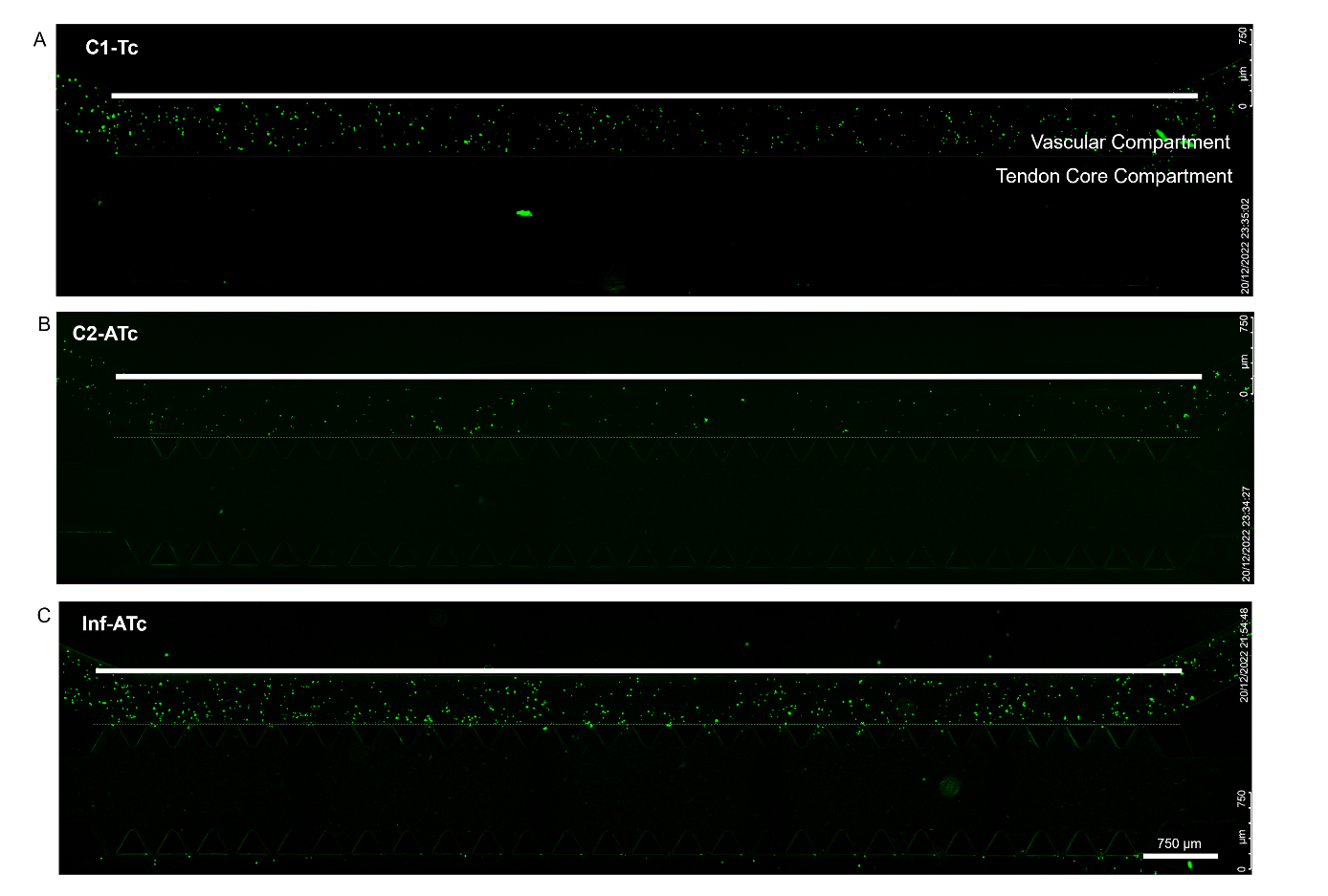


Figure S8: Tile scan of endpoint of timelapse experiment after 24 hrs A) non stimulated hTDCs with non activated T cells (C1-Tc), B) non stimulated hTDCs with activated T cells (C2-ATc) C) hTDCs stimulated with IL-1β and acitivated T cells (Inf-ATc) show extravasation of activated T cells. (scale bar: 750 µm).


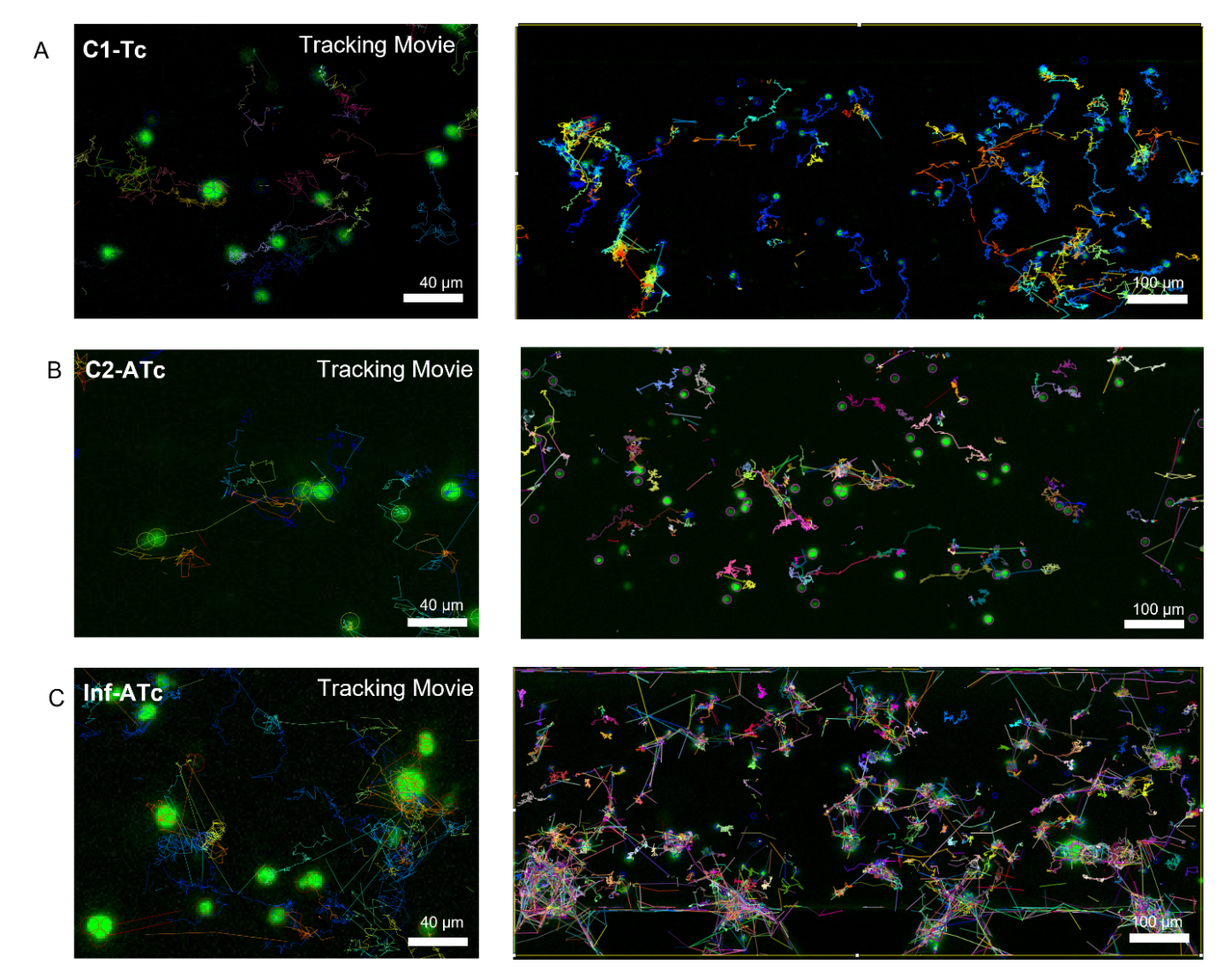


Figure S9: Automatic cell tracking using TrackMate plugin. The colour of trackes represents increasing total distance from blue to red. TrackMate can resolve individual tracks for cells that migrate through crowded regions. Videos of the tracked image stacks shown in avaible in supporting materials.


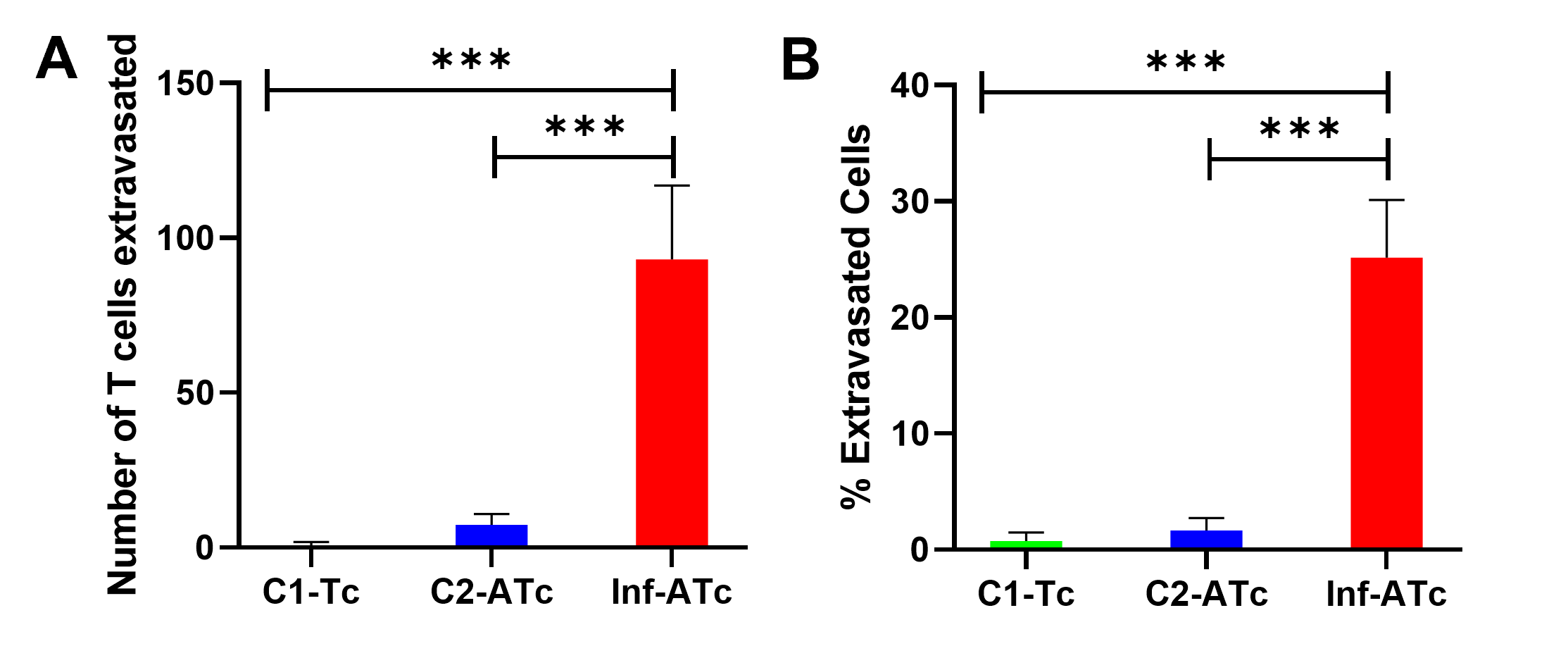


Figure S10: Quantification of T cell number extravasated into intrinsic compartment after 24 hrs in culture. A) Number of T cells, B) % of T cell extravasated into core compartment. Statistical difference ****p* < 0.001 determined by one-way ANOVA followed by Bartlett's test.

Table 1: List of genes evaluated for real time PCR (F: forward primer; R: reverse primer).

| **Target gene** | **Primer sequence**  **Forward**  **Reverse** |
| --- | --- |
| Glyceraldehyde 3- phosphate dehydrogenase GAPDH | AGCCTCAAGATCATCAGCAA GTCATGAGTCCTTCCAGGAT |
| Collagen I, α1  COL-I | CCTGACGCACGGCCAAGAGG  GGCAGGGCTCGGGTTTCCAC |
| Collagen III, α1  COL-III | TTGGCATGGTTCTGGCTTCC GCTGGCTACTTCTCGTG |
| Decorin  DNC | CAGCATTCCTCAAGGTCTTCCT  GAGAGCCATTGTCAACAGCA |
| Tenascin  TNC | ACTGCCAAGTTCACAACAGACC CCCACAATGACTTCCTTGACTG |
| Tenomodulin  TNMD | CCGCGTCTGTGAACCTTTAC CACCCACCAGTTACAAGGCA |
| Scleraxis transcription factor  SCX | AGAACACCCAGCCCAAACAGAT TCGCGGTCCTTGCTCAACTTT |
| Interleukin-6  IL-6 | AGGAGACTTGCCTGGTGAAA  GCATTTGTGGTTGGGTCAG |
| Interleukin-8  IL-8 | GGTGCAGTTTTGCCAAG  TTCCTTGGGGTCCAGACAGA |
| C-C motif chemokine ligand 2  CCL-2 | ACAAGCAAACCCAAACTCCG  AACAGGGTGTCTGGGGAAAG |

**Supplementary Movies:**

Timelapse extravasation of activated T cells

S 1: C1-Tc (Green Channel)

S 2: C2-ATc(Green Channel)

S 3: Inf-ATc (Green Channel)

Videos for automatic cell tracking with TrackMate Image J plugin

S 4: C1-Tc

S 5: C2-ATc

S 6: Inf-ATc
